# Changes in long non-coding RNA expression profiles related to the antagonistic effects of *Escherichia coli* F17 on lamb spleens

**DOI:** 10.1038/s41598-018-34291-0

**Published:** 2018-11-08

**Authors:** Chengyan Jin, Jianjun Bao, Yue Wang, Weihao Chen, Tianyi Wu, Lihong Wang, Xiaoyang Lv, Wen Gao, Buzhong Wang, Guoqiang Zhu, Guojun Dai, Wei Sun

**Affiliations:** 1 Key Laboratory for Animal Genetics, Breeding, Reproduction and Molecular Design of Jiangsu Province, College of Animal Science and Technology, Yangzhou University, Yangzhou, 225009 Jiangsu P. R. China; 2 Jiangsu Xilaiyuan Ecological Agriculture Co., Ltd. Taizhou, 225300 Jiangsu, P. R. China; 3 College of Veterinary Medicine, Yangzhou University, Yangzhou, 225009 Jiangsu P. R. China; 4 Joint international research laboratory of agriculture and agri - product safety of Ministry of Education of China, Yangzhou University, Yangzhou, 225009 Jiangsu P. R. China

## Abstract

Sheep colibacillosis is one of the most common bacterial diseases found at large-scale sheep farms. The aim of this study was to employ RNA-seq to screen differentially expressed (DE) long non-coding RNAs (lncRNAs) that impart antagonistic or sensitive effects on *Escherichia coli* F17. In this study, individuals who had antagonistic or sensitive responses to *E. coli* F17 were identified by feeding *E. coli* F17 strains to Hu lambs. The sensitive group had higher levels of intestinal bacteria than that in the antagonistic group (P < 0.05), the jejunum showed various levels of mucosal tissue damage and had a dark colour, and disintegration of part of the small intestinal villi was observed. Totals of 34 DE lncRNAs and 703 DE mRNAs in two groups were identified. qRT-PCR results for 12 randomly selected DE lncRNAs and DE mRNAs were consistent with the RNA-seq data. Gene Ontology (GO), KEGG Pathway enrichment and lncRNA-mRNA interaction analyses identified 6 co-expressed genes, namely, *MYO1G*, *TIMM29*, *CARM1*, *ADGRB1*, *SEPT4*, and *DESI2*. This is the first study that has performed expression profiling of lncRNAs in the spleen of antagonistic and sensitive lambs. The identification of DE lncRNAs can facilitate investigations into the molecular mechanism underlying resistance to diarrhoea in sheep.

## Introduction

Sheep colibacillosis is one of the most common bacterial diseases found at large-scale sheep farms. The traditional method of controlling the bacterial disease is by antibiotic therapy, although this approach also has several disadvantages. Detecting the expression of antagonistic genes in sheep colibacillosis provides information that may facilitate the elucidation of the molecular mechanism underlying disease resistance to *Escherichia coli*. In 1966, Orskov *et al*.^1^ first reported the porcine *E. coli* adhesion antigen K88, which is an episome-determined antigen^2^. In addition, the morphology of the pilus situated on the surface of the bacteria has been examined by electron microscopy. To date, numerous animal-derived enterotoxigenic *Escherichia coli* (ETEC) pili have been identified, including K88, K99, 987P, F17 and F41, which are all vital virulence factors. Long non-coding RNAs (lncRNAs) are a class of non-coding RNAs longer than 200 nucleotides. Several studies have revealed that lncRNAs are closely related to the development of human tumours, cardiovascular diseases, and metabolic diseases. Furthermore, there is growing evidence that lncRNAs play a significant regulatory role in anti-viral and other natural immune responses^3–7^. However, research on the function of lncRNAs has mainly focused on the regulation of muscle growth, testicular development, hair follicle development, and other traits in sheep, which are less well studied compared to humans^8–10^. A few studies have recently focused on disease prevention and control in sheep^11,12^, including disease resistance, yet our understanding of its underlying molecular mechanism is limited. In the present study, the expression levels of lncRNAs in two sheep spleen phenotypes, antagonistic or sensitive to pili of *E. coli* F17, were assessed by RNA-seq, and key lncRNAs were investigated by target prediction and functional annotation analysis based on the *cis* mechanism, a transcription activation and expression control method for adjacent mRNAs by non-coding RNAs. The results were further verified by q-PCR. Our results provide a deeper understanding of sheep antagonism to *E. coli* F17 in terms of lncRNAs, facilitate in the identification of additional functional genes that are antagonistic to *E. coli* F17, and provide a theoretical basis for solving key issues that are related to breeding indigenous disease-resistant sheep in China.

## Results

### Histological observation and comparison of the number of intestinal bacteria in the antagonistic and sensitive groups

**Figure 1:**
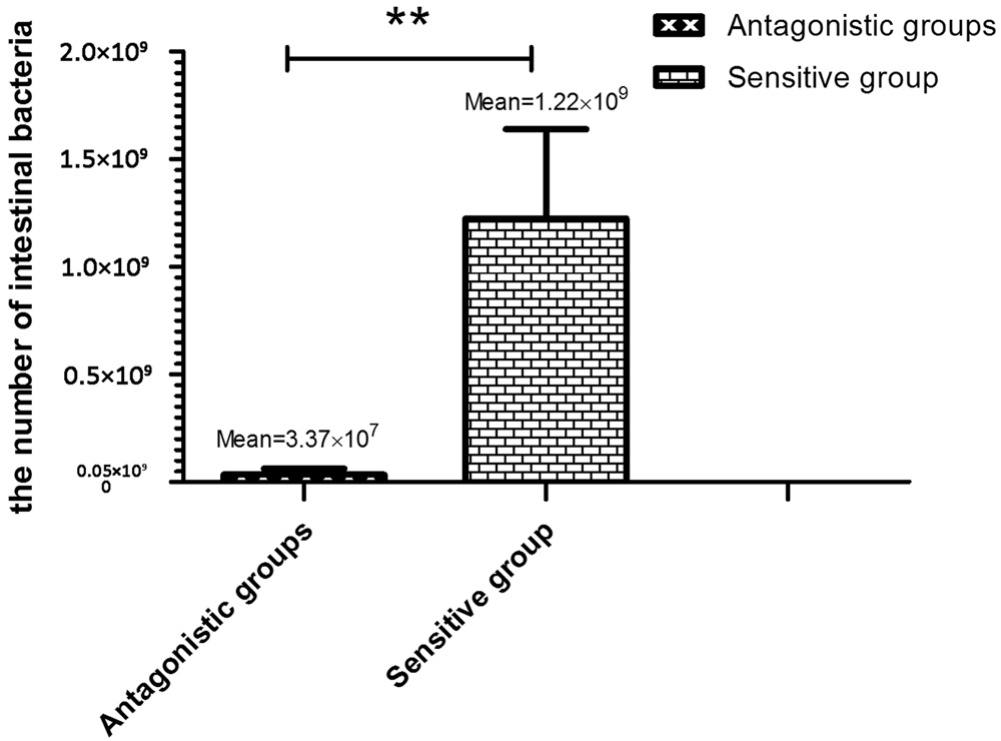
Difference analysis of the intestinal bacteria in antagonistic and sensitive lambs.

**Figure 2:**
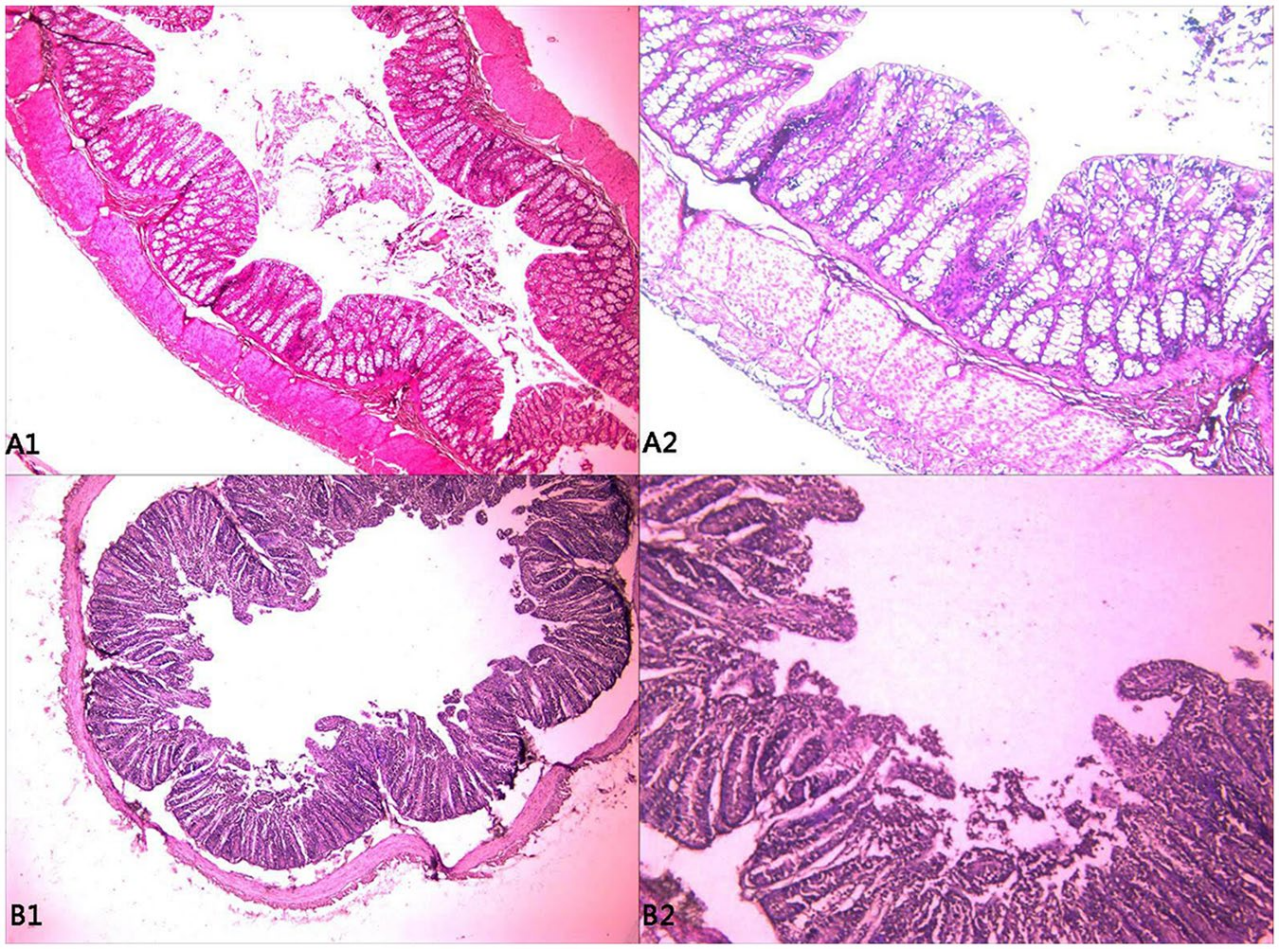
Histological assessment of the jejunum of the antagonistic (**A1**, **A2**) and sensitive (**B1**, **B2**) groups. (**A1** and **B1**) are 200x microscope observations, (**A2** and **B2**) are 400x microscope observations.

Based on faecal morphology^13^, the experimental subjects were divided into the antagonistic group (12p, 13p, 14p) and the sensitive group (15p, 16p, 17p). In the sensitive groups, the bacterial count was arranged from 4.7*10^8^ to 1.9*10^9^, the mean of the bacterial count was 1.22*10^9^, and yet the count dropped 5.1*10^6^ to 9.0*10^7^ in the antagonistic groups, the mean of the bacterial count was 3.37*10^7^ (Table 1). Compared to the antagonistic group, the number of intestinal bacteria in the sensitive group was significantly higher (*P* < 0.05, Fig. 1). The jejunum mucosal tissues of lambs in the sensitive group were damaged, dull in colour, lysed, and exhibited large lacuna that could be observed at the submucosal layer. The intestinal villi had disintegrated and were highly vascularized (i.e., capillaries), and the intestinal mucosa showed severe damage, thus making it difficult to prepare histological sections for assessment (Fig. 2).

**Table 1:** Comparison of the number of intestinal bacteria between the antagonistic and sensitive lambs.

Group	Intestinal tract	Dilution factor				Bacterial count (CFU·mL^−1^)
		10^5^	10^6^	10^7^	10^8^	
Antagonistic groups	Duodenum	70	6	ng	ng	6.0 × 10^6^
	Jejunum	51	ng	ng	ng	5.1 × 10^6^
	Ileum	>500	170	9	ng	9.0 × 10^7^
Sensitive group	Duodenum	>1,000	>500	128	13	1.3 × 10^9^
	Jejunum	>1,000	88	47	ng	4.7 × 10^8^
	Ileum	>1,000	>500	176	19	1.9 × 10^9^

Note: “ng” means “no growth”.

### Summary of RNA sequencing of spleens in sheep

cDNA libraries of the lamb spleens from the antagonistic and sensitive groups were constructed. Sequencing was performed using the Illumina HiSeq 2500 platform. The antagonistic and sensitive groups generated a total of 354,943,820 and 370,616,990 raw reads, with GC contents of 48.33% and 49.67%, respectively. The valid reads in the clean reads were mapped to the *O. aries* v4.0 reference genome, and more than 73.5% of the reads were mapped to the genome. The reads mapped to multiple locations of the reference sequence were less than 4.5% and more than 70% of the reads were uniquely mapped to the reference sequence. Approximately 35% of the reads mapped to the positive and negative chains of the genome. In addition, the number of reads that were mapped to the exonic regions (~60%) was higher than those that were mapped to intergenic and intron regions by annotation analysis. These results indicate that the matching efficiency of our *de novo* assembly is high, and most reads mapped to the exonic region (Table 2).

**Table 2:** Read statistics of the reference genome.

Sample	Sample_12p	Sample_13p	Sample_14p	Sample_15P	Sample_16p	Sample_17P
Raw reads	135,067,560	100,031,546	119,844,714	110,990,304	122,657,326	136,969,360
Total reads	101,734,214	76,072,598	82,901,000	82,165,530	88,819,554	93,869,100
Total number of reads mapped	78,625,843 (77.29%)	58,935,848 (77.47%)	62,008,068 (74.80%)	60,455,546 (73.58%)	67,337,265 (75.81%)	69,827,122 (74.39%)
Number of reads that mapped to multiple regions	3,792,793 (3.73%)	2,606,663 (3.43%)	3,322,008 (4.01%)	3,470,863 (4.22%)	3,937,078 (4.43%)	3,997,927 (4.26%)
Number of reads that were mapped to unique regions	74,833,050 (73.56%)	56,329,185 (74.05%)	58,686,060 (70.79%)	56,984,683 (69.35%)	63,400,187 (71.38%)	65,829,195 (70.13%)
Read-1	38,037,308 (37.39%)	286,21,024 (37.62%)	29,752,817 (35.89%)	28,436,715 (34.61%)	32,177,909 (36.23%)	33,403,729 (35.59%)
Read-2	36,795,742 (36.17%)	27,708,161 (36.42%)	28,933,243 (34.90%)	28,547,968 (34.74%)	31,222,278 (35.15%)	32,425,466 (34.54%)
Number of reads that map to ‘+’	37,435,639 (36.80%)	28,177,639 (37.04%)	29,369,825 (35.43%)	28,554,241 (34.75%)	31,731,741 (35.73%)	32,953,615 (35.11%)
Number of reads map to ‘−’	37,397,411 (36.76%)	28,151,546 (37.01%)	29,16,235 (35.36%)	28,430,442 (34.60%)	31,668,446 (35.65%)	32,875,580 (35.02%)
Number of non-splice reads	65,956,768 (64.83%)	50,126,819 (65.89%)	50,492,679 (60.91%)	48,280,120 (58.76%)	54,077,425 (60.88%)	56,555,536 (60.25%)
Number of splice reads	8,876,282 (8.72%)	6,202,366 (8.15%)	8,193,381 (9.88%)	8,704,563 (10.59%)	9,322,762 (10.50%)	9,273659 (9.88%)
Number of reads mapped in proper pairs	66,749,602 (65.61%)	50,161,962 (65.94%)	51,970,094 (62.69%)	51,198,774 (62.31%)	56,791,332 (63.94%)	58,971,888 (62.82%)

### Identification of transcripts in sheep spleens

**Figure 3:**
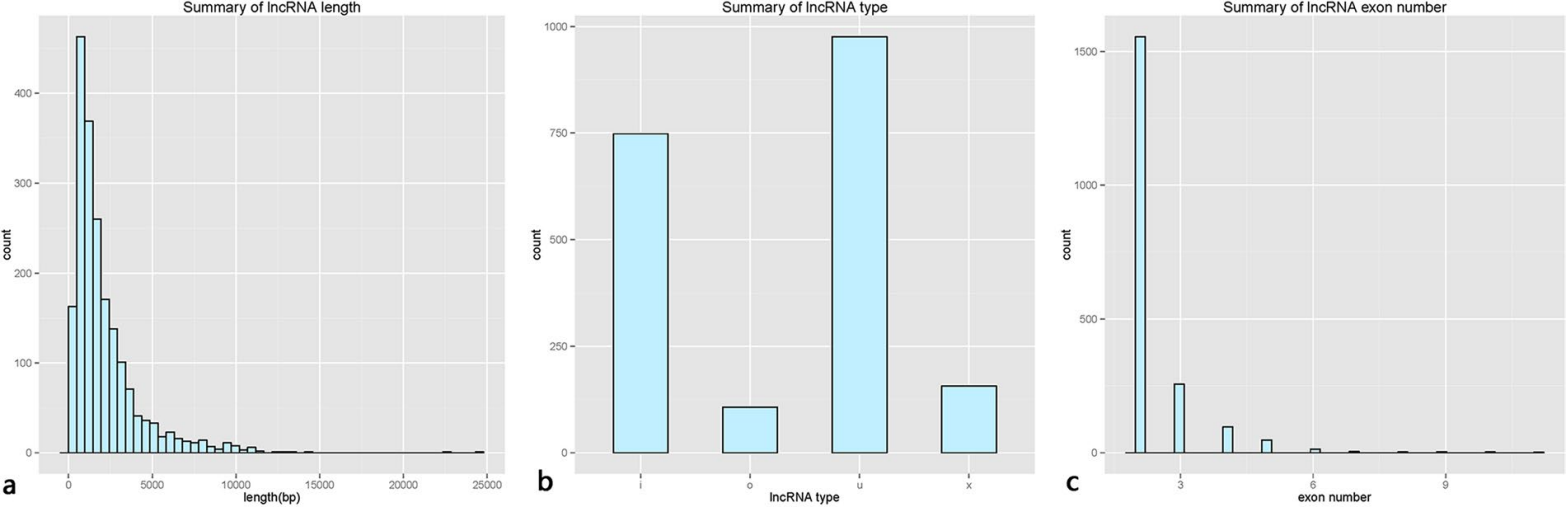
Summary of the lengths, types, and number of exons of the predicted lncRNAs.

After mapping the reference sequence, we identified 1,988 lncRNAs and 38,843 mRNAs from 42,460 compiled transcripts. The length of the lncRNAs was mainly distributed within the range of 200 bp-5,000 bp (Fig. 3a), and the average length was 2,124 bp. Additionally, the lncRNA types mainly include intergenic lncRNAs (character u) (Fig. 3b)and intronic lncRNAs (character i), containing 2 to 3 exons (Fig. 3c).

### Analysis and validation of DE transcripts

**Figure 4:**
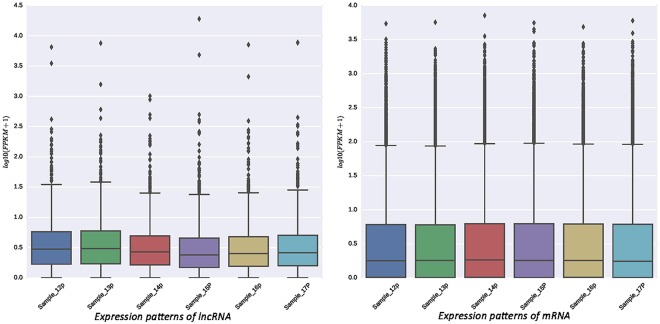
Expression patterns of lncRNA and mRNA transcripts. The Box-whisker Plot consists of five statistics: the minimum, the first quartile (25%), the median (50%), the third quartile (75%), and the maximum.

**Figure 5:**
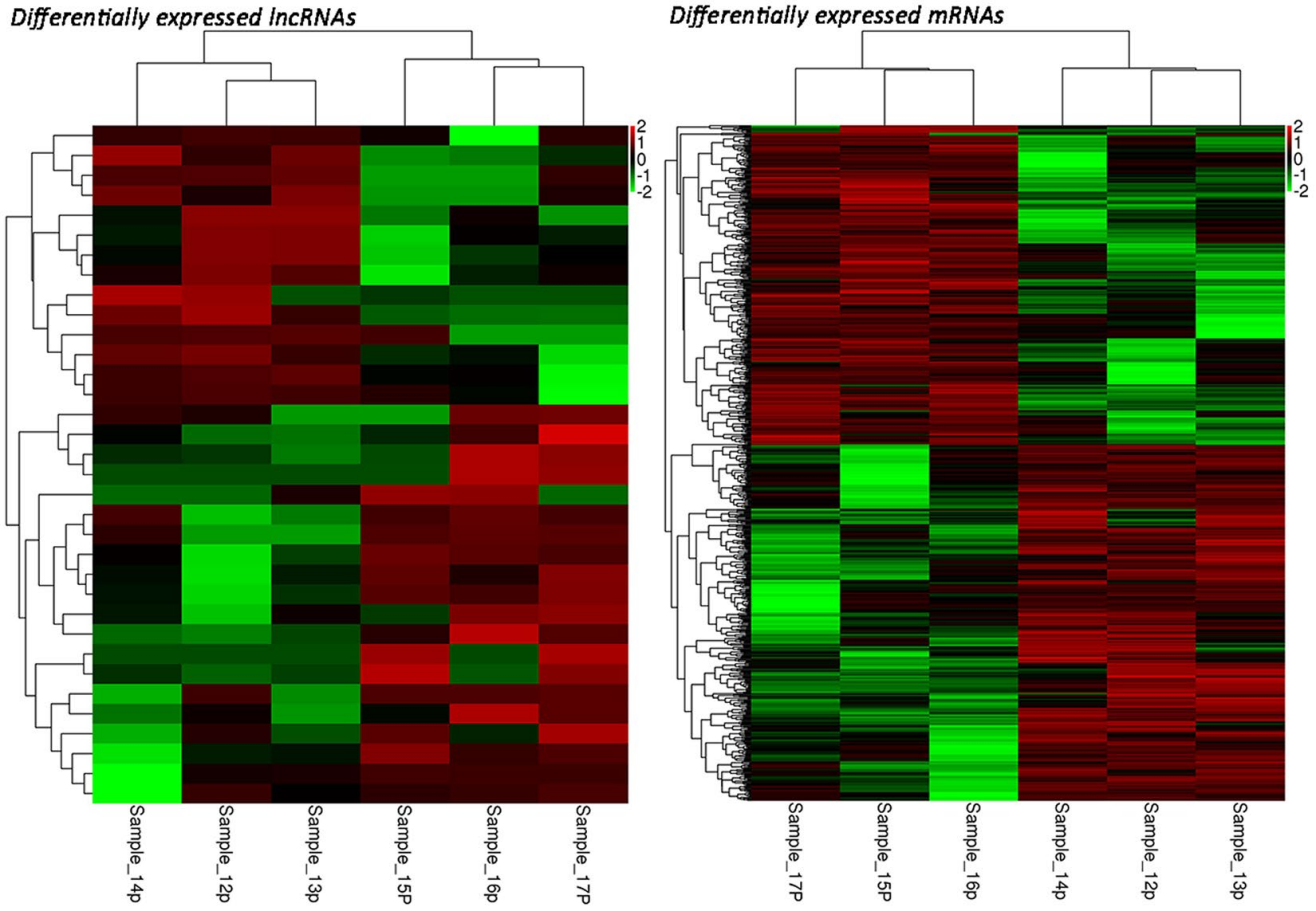
Differentially expressed lncRNAs and mRNAs between antagonistic and sensitive lambs. Color indicates the amount of expression of the gene. The darker the color, the greater the expression (red is up-regulated, green is down-regulated). Each row represents the expression of each gene in different samples, and each column represents the expression of all genes in each sample. The top tree shows the results of cluster analysis of different samples from different experimental groups, and the left tree shows the results of cluster analysis of different genes from different samples.

**Figure 6:**
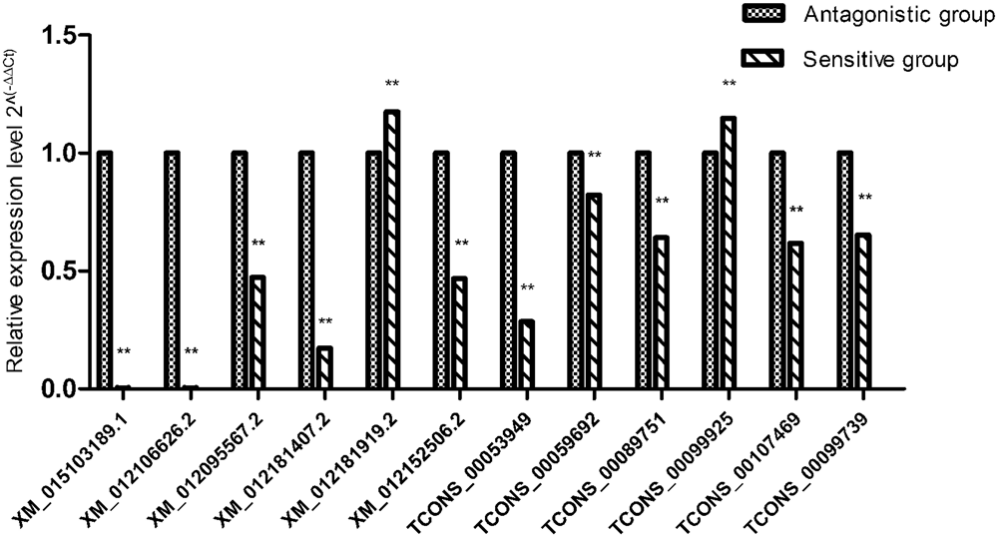
Relative expression levels of DE lncRNAs and mRNAs between antagonistic and sensitive lambs were confirmed by q-PCR. Note: “**” means highly significant correlation; “*” means significant correlation; “ns” or “no SuperiorScript” means no significant correlation. The same as below.

**Figure 7:**
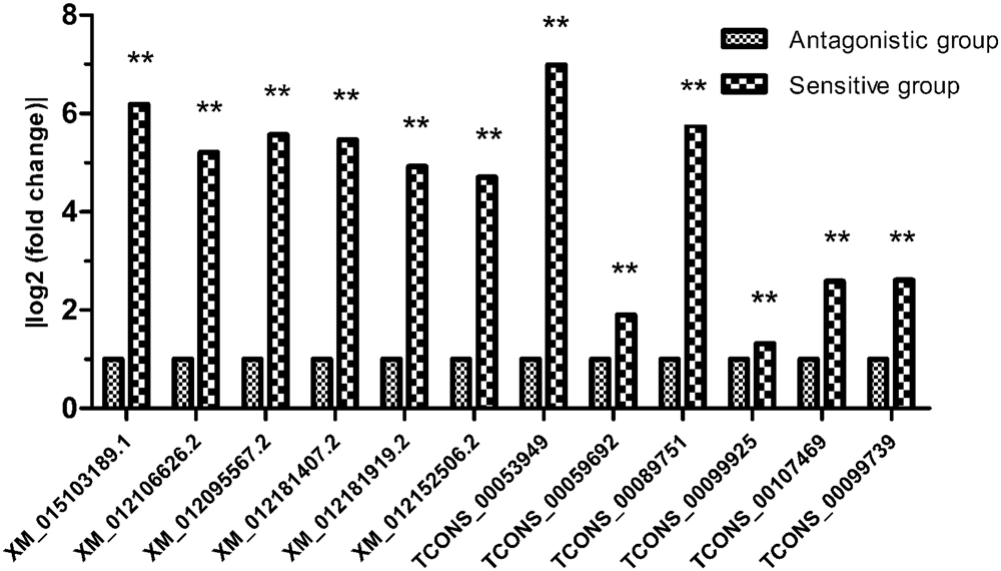
RNA-seq results of DE lncRNAs and mRNAs.

The expression levels of lncRNA and mRNA transcripts were estimated using the FPKM values. We found that the expression level of the lncRNA transcripts was relatively low (Fig. 4). A total of 14 upregulated and 20 downregulated DE lncRNAs and 370 upregulated and 333 downregulated DE mRNAs were screened under conditions of P < 0.05 and |log_2_ (fold change)| > 1 (Fig. 5). To further verify the reliability of our RNA-seq data, a total of 12 DE lncRNAs and DE mRNAs were randomly selected. Their relative expression levels in the antagonistic and sensitive lambs were confirmed by q-PCR (Fig. 6) and were found to coincide with our RNA-seq results (Fig. 7), thus indicating that our RNA-seq data were reliable. Our analyses also showed that high-throughput sequencing has the advantage of detecting genes that are expressed at relatively very low levels (0 < FPKM < 1).

### GO and KEGG Pathway enrichment analysis of DE lncRNAs

**Figure 8:**
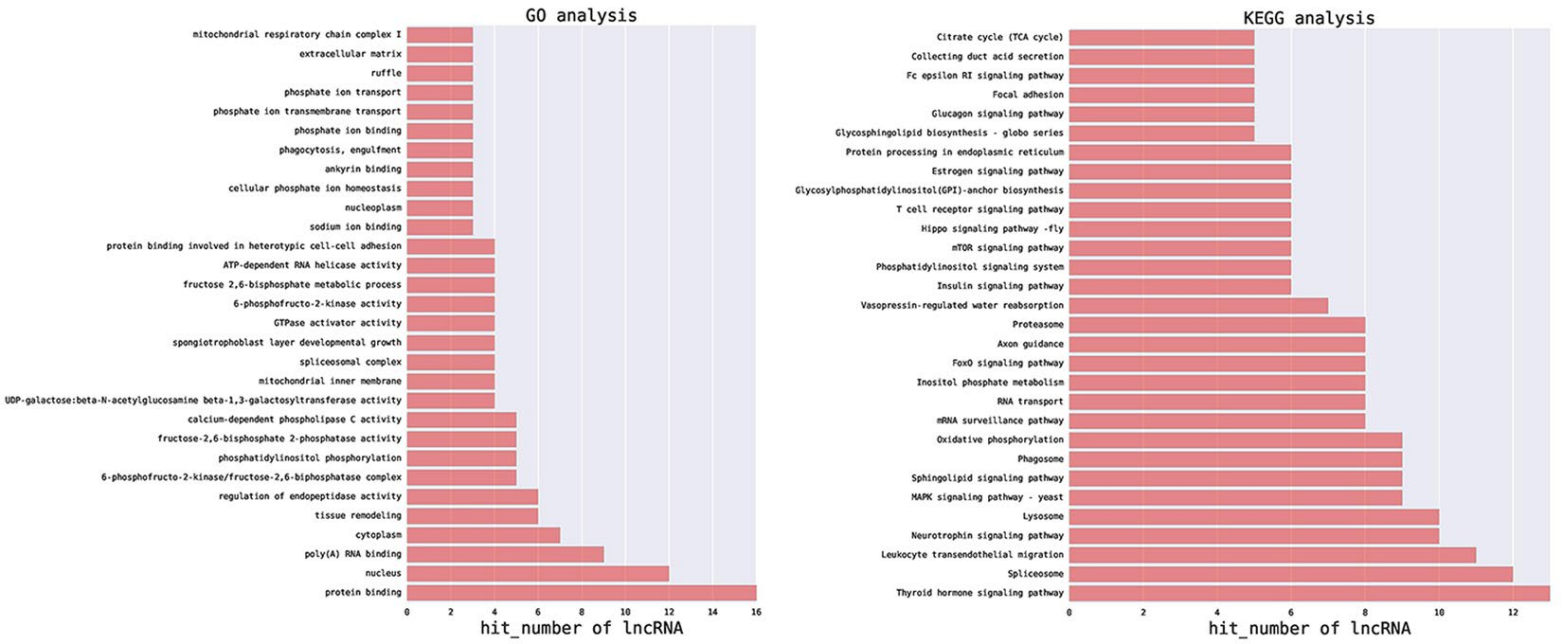
Gene Ontology and KEGG Pathway enrichment analyses of DE lncRNAs.

In the corresponding relationship between “lncRNA name-function prediction Term” (Supplementary 1 and Supplementary 2), we selected the top 500 predictive relationships with the highest predictive reliability (sorted by p-value). The frequency of each function and the number of GO (or pathway) terms with function annotations were analysed to reflect the difference in the distribution of lncRNAs (Fig. 8).

Comparisons of the DE lncRNAs and the entries in the GO database identified that a total of 34 lncRNAs could be annotated and classified into 302 functional subclasses. The number of DE lncRNAs in the top 30 functional subclasses is shown Fig. 8. The number of DE lncRNAs in the categories of protein binding (GO:0005515), nucleus (GO:0005634), poly(A) RNA-binding (GO:0044822), cytoplasm (GO:0005737), tissue remodelling (GO:0048771), regulation of endopeptidase activity (GO:0052548, 6-phosphofructo-2-kinase/fructose-2,6-biphosphatase complex (GO:0043540), phosphatidylinositol phosphorylation (GO:0046854), fructose-2,6-bisphosphate 2-phosphatase activity (GO:0004331), and calcium-dependent phospholipase C activity (GO:0050429) were higher.

Comparison between the DE lncRNAs and the entries in the KEGG Pathway database indicated that a total of 34 lncRNAs could be annotated and classified into 149 KEGG pathways. The number of DE lncRNAs in the top 30 KEGG Pathways is shown in Fig. 8. The number of DE lncRNAs in the thyroid hormone signalling pathway (path:ko04919), spliceosome (path:ko03040), leukocyte transendothelial migration (path:ko04670), neurotrophin signalling pathway (path:ko04722), lysosome (path:ko04142), MAPK signalling pathway-yeast (path:ko04011), sphingolipid signalling pathway (path:ko04071), phagosome (path:ko04145), and oxidative phosphorylation (path:ko00190) categories were relatively higher.

### LncRNAs and their adjacent coding genes

We searched for all the coding genes within the 100-kb flanking regions of the lncRNAs, and genes that were significantly co-expressed with the lncRNAs as indicated by Pearson correlation calculations were identified. These co-expressed genes that were adjacent to the lncRNAs were presumed to be regulated by lncRNAs. Therefore, we identified 6 genes may be regulated by their associated lncRNAs (Table 3).

**Table 3:** DE lncRNAs and their co-expressed gene.

lncRNA	Change in expression	mRNA	Gene	Regulation	Chromosome	Pearson p-value	Pearson correlation	*Cis* distance (bp)
TCONS_00053949	Downregulated	XM_012176963.2	MYO1G	Up	NC_019461.2	0.034	−0.845	1,670
TCONS_00059692	Upregulated	XM_015095635.1	TIMM29	Down	NC_019462.2	0.01	−0.916	16,037
TCONS_00059692	Upregulated	XM_015095835.1	CARM1	Down	NC_019462.2	0.033	−0.848	22,513
TCONS_00089751	Upregulated	XM_015097644.1	ADGRB1	Up	NC_019466.2	0.0027	0.957	8,560
TCONS_00099925	Downregulated	XM_015098457.1	SEPT4	Up	NC_019468.2	0.049	−0.812	98,788
TCONS_00107469	Upregulated	XM_012187185.1	DESI2	Up	NC_019469.2	0.0026	0.958	94,714

## Discussion

Due to the rapid development of transcriptome analysis, lncRNAs have recently been considered as a novel modulator of cell development^14^. The lncRNAs that we have identified in this study are mainly associated with cancer, such as prostate cancer^15^, gastric cancer^16^, lung cancer^17^ and breast cancer^18^, as well as reproduction^19–22^. However, investigations of lamb diarrhoea in relation to lncRNAs are limited. The Hu sheep is a unique breed with high fecundity and strong adaptability to warm-wet climates and can be kept indoors all year. This study has provided the first overview of lncRNAs in relation to diarrhoea in sheep, as well an investigation into their possible roles in disease resistance.

Major economic losses in sheep farms are often due to diarrhoea. In this study, We mainly study the immune status of lambs, and the spleen is the largest immune organ; thus, we selected the spleen as the research object. we found that the expression level of lncRNAs was lower than mRNAs in lamb spleens (Fig. 4), which was in agreement with the results from sheep testicular tissues^9^, and the average lengths of lncRNAs and mRNAs in sheep were longer than those in pigs (1,713 bp and 1,983 bp, respectively)^20^. We searched for all the coding genes within the 100-kb flanking regions of the lncRNAs and identified intersecting genes that were significantly co-expressed with the lncRNAs as indicated by Pearson correlation calculations^23^. We also identified the following 6 genes as being co-expressed with the lncRNAs: myosin IG (*MYO1G*), translocase of inner mitochondrial membrane 29 (*TIMM29*), co-activator associated arginine methyltransferase 1 (*CARM1*), adhesion G protein-coupled receptor B1 (*ADGRB1*), septin 4 (*SEPT4*), and desumoylating isopeptidase 2 (*DESI2*).

*MYO1G* plays an important role in maintaining cell stiffness in B-cell lymphocytes. The deletion of the myo1g gene results in a reduction in cell stiffness, which in turn affects cell adhesion, proliferation, phagocytosis, and endocytosis in B-cell lymphocytes^24^. Investigations of *TIMM29* are limited. *TIMM29* has been identified as the first specific component of the mammalian *TIMM22* protein complex and plays an important role in the assembly of the *TIMM23* protein^25,26^. *CARM1*, a member of the protein arginine methyltransferase (*PRMT*) family, is an enzyme with a highly conserved domain with methyltransferase activity. *CARM1* knockout mice die at birth^27^, indicating that *CARM1* is essential to postnatal survival. It was later discovered that *CARM1* inhibition promotes HIV-1 activation^28^. *ADGRB1* is a member of the transmembrane protein-adhesion G protein coupled receptor (aGPCR) family, which is characterized by a conserved GAIN domain that has autologous proteolytic activity that can cleave the receptor near the first transmembrane domain. Studies have shown that the new N-terminal stalk, which is revealed by GAIN domain cleavage, can directly activate aGPCRs as a tethered agonist^29^. Septins are a highly conserved cytoskeletal family with GTPase activity. The tumour suppressor *SEPT4* is a member of the septin family that can induce cancer cell apoptosis^30^. Mutations in the *SEPT4* gene in mice can lead to disorders involving the annuli of spermatozoa^31^ and adjacent cortical structures, thereby causing low sperm motility, ultimately leading to infertility^32^. *DESI2* is a pro-apoptotic gene; *in vitro* experiments have shown that its overexpression induces apoptosis in pancreatic cancer and other tumour cells, which can effectively inhibit the proliferation of some cancer cells. Gene therapy using *DESI2* and *IP10* significantly inhibits tumour growth and effectively prolongs the survival of tumour-bearing mice^33,34^.

A total of 703 mRNAs and 34 known lncRNAs were differentially expressed between the antagonistic and sensitive groups, including 14 upregulated lncRNAs and 20 downregulated lncRNAs. In addition, the present study identified 1,942 novel lncRNAs. We searched for all the coding genes within the 100-kb flanking regions of the lncRNAs and identified genes shared between the two experimental groups that were significantly co-expressed with lncRNAs as indicated by Pearson correlation calculations. We have determined that 6 genes may be regulated by their associated lncRNAs. To validate our RNA-seq results, q-PCR was performed to verify the expression levels of the 12 known lncRNAs and mRNAs. The final results coincided with our RNA-seq data.

GO is a bioinformatics tool that has been extensively utilized in studying the relationship of various gene functions. GO analysis indicated that 16 out the 34 DE lncRNAs were enriched with the protein binding (GO: 0005515) category. Moreover, KEGG Pathway analysis showed that the sphingolipid signalling (path: ko04071), axon guidance (path: ko04360), and glycosylphosphatidylinositol (GPI)-anchor biosynthesis (path: ko00563) pathways may be important KEGG pathways of genes co-expressed with DE lncRNAs, and the related lncRNAs may be potentially involved in fimbriae adhesion to the intestinal mucosa. However, the role of these pathways in disease resistance remains largely unknown.

In this study, the expression profiles of lncRNAs in the spleens of lambs that were antagonistic or sensitive to diarrhoea were investigated to further understand the regulation of lncRNAs in sheep disease resistance. Several differentially expressed lncRNAs in lamb spleens between the antagonistic and sensitive groups were identified, and we found that 6 genes (*MYO1G*, *TIMM29*, *CARM1*, *ADGRB1*, *SEPT4*, and *DESI2*) may be regulated by their associated lncRNA. Our study may help elucidate the mechanism underlying resistance to diarrhoea in lambs. Further investigations of these sheep lncRNAs in relation to diarrhoea-resistance are warranted.

## Methods

### Ethics statement

The Institutional Animal Care and Use Committee (IACUC) of the government of Jiangsu Province (Permit Number 45) and the Ministry of Agriculture of China (Permit Number 39) approved the animal study proposal. All experimental procedures were conducted in strict compliance with the recommendations of the Guide for the Care and Use of Laboratory Animals of Jiangsu Province and of the Animal Care and Use Committee of the Chinese Ministry of Agriculture. All efforts were made to minimize animal suffering.

### Experimental design and sample collection

The experimental sheep were purchased from Jiangsu Xilaiyuan Ecological Agriculture Co., Ltd. in December 2016. A total of 18 three-day-old lambs showing normal growth and approximately similar weight were randomly selected, and all the sheep were raised with segregation. To ensure their dietary requirements, the sheep were fed with 10% lamb milk powder (Table 4) prior to the experiment. Five-day-old lambs were fed 12.5% lamb milk powder and *E. coli* F17 bacteria liquid [4.6 × 10^8^ colony-forming units (CFUs)·mL^−1^], as well as *ad libitum* access to drinking water. Stool features of the experimental lambs were recorded daily (Table 5). Lambs that exhibited diarrhoea for two days were classified as antagonistic and sensitive and were euthanized. The intestinal tissues were collected in 4% paraformaldehyde. The liver, spleen, duodenum, jejunum, and ileum of each lamb were collected and immediately frozen in liquid nitrogen until RNA extraction.

**Table 4:** Lamb Milk Powder Ingredients List (per 100 grams of milk powder).

Components	Moisture content	Crude protein	Crude fat	Crude fibre	Coarse ash	Calcium	Total phosphorus	Lysine
Percentage	≤6	≥22	≥18	≤5	≤8	0.9~3.0	≥0.5	≥1.0

**Table 5:** Bristol Stool Form Scale^13^.

Type 1	Separate hard lumps, similar to nuts
Type 2	Sausage-shaped but lumpy
Type 3	Sausage- or snake-like but with cracks on its surface
Type 4	Sausage- or snake-like, smooth, and soft
Type 5	Soft blobs with distinct edges
Type 6	Fluffy pieces with ragged edges, mushy stool
Type 7	Watery, no solid pieces

### HE staining

The jejunum tissue was washed with 0.9% normal saline and fixed in 4% paraformaldehyde for 48 h at room temperature and then used in histological analysis. Next, 7 μm-thick sections were stained with haematoxylin-eosin and the morphology of the jejunum epithelia was assessed under a microscope.

### Library construction and sequencing

RNA was extracted from the spleen of three individuals from each group. A NanoDrop 2000 Ultra Microscope and an Agilent 2100 Bioanalyzer were utilized in determining the quality control of the extracted total RNAs (Annex 1). Ribosomal RNA was removed using a Ribo-Zero (TM) kit (Epicenter, Madison, WI, USA). Short fragments (approximately 200 bp in length) were obtained and used as templates for first-stand cDNA synthesis. Second-strand cDNA synthesis was performed using a buffer, dNTPs, Rnase H, and DNA polymerase I. After PCR amplification and purification using the Qubit® dsDNA HS Assay Kit, the cDNA library was constructed using an NEBNext® Ultra™ RNA Library Preparation Kit. The cDNA library was sequenced on the Illumina HiSeq 2500 platform at Shanghai OE Biomedical Technology Co. (sequencing read length: 150 bp).

### Identification of lncRNAs and mRNAs

The raw data were filtered to eliminate low-quality reads. Clean reads mapped to the reference genome (*Ovis aries* v4.0) were selected for *de novo* assembly. Coding and non-coding RNA candidates from the unknown transcripts were categorized using four coding potential analysis methods, CPC^35^, CNCI^36^, Pfam^37^, and PLEK^38^. The minimum length and the number of exons were set as thresholds, thereby filtering putative encoded RNAs, and transcripts containing two exons and longer than 200 nt were selected as candidate lncRNAs. Different types of lncRNAs were classified by cuffcompare, including intergenic lncRNAs (character u), intronic lncRNAs (character i), anti-sense lncRNAs (character x), and sense-overlapping lncRNAs (character o).

### Different expression analysis

Because the fragments per kb per million reads (FPKM) method^39^ considers the simultaneous effect of sequencing depth and the length of the transcript on the number of fragments, the FPKM value was used to estimate the expression levels of lncRNA and mRNA transcripts. We used DESeq^40^ to determine the number of DE genes and the FPKM values between the two groups. In cases when RNA-seq data were employed to compare different expression levels in the same transcript in both samples, two criteria were selected: 1) fold change, which is the change in the expression level of the same transcript in both samples; and 2) the p value or false discovery rate (FDR) (adjusted p-value). The FDR error control method was used in correcting the p-value multiple hypothesis test^41^.

### GO and KEGG pathway analyses

After screening for differentially expressed transcripts, functional annotation was performed using GO enrichment analysis. Enrichment analysis employed counting the number of transcripts in each GO term, followed by Fisher’s exact test to assess statistical significance (p < 0.05). KEGG^42^ is the main public database used in pathway analysis, which was followed by Fisher’s exact test to assess statistical significance (p < 0.05). The analysis of different transcripts was used to identify enriched pathways.

### Prediction of the target genes of DE lncRNAs

The target genes of DE lncRNAs were predicted by calculating the Pearson correlation coefficients and P values among multiple genes. The |correlation| ≥ 0.7 and P ≤ 0.05 were used to filter the transcripts^43^. The DE lncRNAs associated with adhesion were selected, and the target genes of all DE lncRNAs were predicted by *cis*-acting^44^.

### Verification of the expression level of DE lncRNAs

To verify whether the screened DE lncRNAs play a role in the process of antagonism, q-PCR was used to detect the expression levels of 12 DE lncRNAs and DE mRNAs in the lamb spleens between the antagonistic and sensitive groups. The relative expression of each RNA was normalized to that of GAPDH using the 2^−∆∆Ct^ method^45^, and the primers used in the amplification of the lncRNAs are shown in Table 6.

**Table 6:** The primers for *GAPDH*, DE lncRNAs, and mRNAs.

	ID	Primer sequence (5′ → 3′)	Product length (bp)
Primers for the six DE mRNAs	*XM_015103189.1*	F: AGCACTTCCTCCTGTCCG	132
		R: CAGCACAGAAGGCAAAGTC	
	*XM_012106626.2*	F: CCGAGTTTGCAGGTACCCAAC	121
		R: TTTTGGCGCATGTATACCTG	
	*XM_012095567.2*	F: TGAGACTCTACTTCGCTGC	102
		R: TTGCCCATCCTTAATAGCTG	
	*XM_012181407.2*	F: ACGACGGTGGTTAAATACTC	125
		R: AGTTGCCCATAGTCACTGGTC	
	*XM_012181919.2*	F: TCAACCATATGCTGACGGAC	119
		R: ATGCCGCCTATCAAGGTC	
	*XM_012152506.2*	F: CCACCTGCGGTTCAAGTTAC	157
		R: TGCCTGAATCACCTTGTC	
Primers for six DE lncRNA	*TCONS_00053949*	F: CCTGGCTATATCCTTACATCAC	100
		R: AAGTTCAAACTCCGCTGCAC	
	*TCONS_00059692*	F: AATTTCTTCTCGTTCCAAGGC	131
		R: CCAACAGGGAGCCAACTTC	
	*TCONS_00089751*	F: AGAAGGCTTTGACCGAAC	153
		R: TCAATGCCCTCCACGAGAC	
	*TCONS_00099925*	F: AGTGCCACATGTACCTAGCAG	146
		R: ACGACAGGCATTTTAACCCATG	
	*TCONS_00107469*	F: TAGTACAGCCCATATTTATCG	115
		R: ATTTTCTTTCCACAGGGACG	
	*TCONS_00099739*	F: CCGACGCTGTCATGATGC	159
		R: TCCGTCTCCAGAACCAAGGC	
Primers for *GAPDH*	*GAPDH*	F: GTTCCACGGCACAGTCAAGG	127
		R: ACTCAGCACCAGCATCACCC	

### Statistical analysis

All data were analysed by SPSS (version 20.0), and the relative expression levels of different transcripts were analysed by ANOVA. Tukey’s test was used for multiple comparisons. Statistical significance was determined when p < 0.05. Each group contained three samples, and each experiment was repeated thrice.

## Electronic supplementary material

Supplementary Information

## Data Availability

We guarantee that our data is valid.
