# Supplementary material for: Changes in long non-coding RNA expression profiles related to the antagonistic effects of Escherichia coli F17 on lamb spleens
Source: Sci Rep. 2018 Nov 8;8:16514. doi: 10.1038/s41598-018-34291-0 (PMC6224397; doi:10.1038/s41598-018-34291-0)
Supplement: Supplementary file 1 — Supplementary Information [file 41598_2018_34291_MOESM1_ESM.pdf]

Title page

1) the title of the manuscript

Changes in long non-coding RNA expression profiles related to the antagonistic effects of Escherichia coli F17 on lamb spleens

2) the author list

Corresponding Author: **Wei Sun**
Company/ Institution: College of Animal Science and Technology, Yangzhou University
Address: 48 Wenhui East Road, Hanjiang District, Yangzhou 225009, Jiangsu, P. R. China.
Contributing Author: **Jianjun Bao**
Company/ Institution: College of Animal Science and Technology, Yangzhou University
Address: 48 Wenhui East Road, Hanjiang District, Yangzhou 225009, Jiangsu, P. R. China.
Contributing Author: **Chengyan Jin**
Company/ Institution: College of Animal Science and Technology, Yangzhou University
Address: 48 Wenhui East Road, Hanjiang District, Yangzhou 225009, Jiangsu, P. R. China.
Contributing Author: **Yue Wang**
Company/ Institution: College of Animal Science and Technology, Yangzhou University
Address: 48 Wenhui East Road, Hanjiang District, Yangzhou 225009, Jiangsu, P. R. China.
Contributing Author: **Weihao Chen**
Company/ Institution: College of Animal Science and Technology, Yangzhou University
Address: 48 Wenhui East Road, Hanjiang District, Yangzhou 225009, Jiangsu, P. R. China.
Contributing Author: **Tianyi Wu**
Company/ Institution: College of Animal Science and Technology, Yangzhou University
Address: 48 Wenhui East Road, Hanjiang District, Yangzhou 225009, Jiangsu, P. R. China.
Contributing Author: **Lihong Wang**
Company/ Institution: College of Animal Science and Technology, Yangzhou University
Address: 48 Wenhui East Road, Hanjiang District, Yangzhou 225009, Jiangsu, P. R. China.
Contributing Author: **Xiaoyang Lv**
Company/ Institution: College of Animal Science and Technology, Yangzhou University
Address: 48 Wenhui East Road, Hanjiang District, Yangzhou 225009, Jiangsu, P. R. China.
Contributing Author: **Wen Gao**
Company/ Institution: College of Animal Science and Technology, Yangzhou University
Address: 48 Wenhui East Road, Hanjiang District, Yangzhou 225009, Jiangsu, P. R. China.
Contributing Author: **Buzhong Wang**
Company/ Institution: Jiangsu Xilaiyuan Ecological Agriculture Co., Ltd.
Address: Dasi Town, Taizhou 225300, Jiangsu, P. R. China.
Contributing Author: **Guoqiang Zhu**
Company/ Institution: College of Veterinary Medicine, Yangzhou University
Address: 48 Wenhui East Road, Hanjiang District, Yangzhou 225009, Jiangsu, P. R. China.
Contributing Author: **Guojun Dai**
Company/ Institution: College of Animal Science and Technology, Yangzhou University
Address: 48 Wenhui East Road, Hanjiang District, Yangzhou 225009, Jiangsu, P. R. China.

3) a short running head at the head of the title page

lncRNA name-function prediction Term

Supplementary 1

lncRNA	Term_ID	P_value	Q_value
TCONS_00059692	GO:0044822	1.09E-10	3.35E-08
TCONS_00083441	GO:0086080	1.16E-08	1.63E-06
TCONS_00083441	GO:0045162	1.63E-08	1.63E-06
TCONS_00083441	GO:0019227	4.78E-08	3.18E-06
TCONS_00107469	GO:0044822	5.03E-08	1.61E-05
TCONS_00066324	GO:0005634	4.66E-07	9.65E-05
TCONS_00083441	GO:0007417	6.18E-07	3.09E-05
TCONS_00083441	GO:0030506	8.00E-07	3.20E-05
TCONS_00116477	GO:0042908	1.32E-06	0.000137047
TCONS_00116477	GO:0046618	1.32E-06	0.000137047
TCONS_00108000	GO:0005634	3.02E-06	0.000716889
TCONS_00000678	GO:0005515	3.08E-06	0.00079127
TCONS_00001295	GO:0044822	3.49E-06	0.000934194
TCONS_00083441	GO:0010975	4.09E-06	0.000136207
TCONS_00099925	GO:0005515	4.65E-06	0.000973186
TCONS_00059692	GO:0005634	4.81E-06	0.000740019
TCONS_00116477	GO:0030522	6.39E-06	0.000289175
TCONS_00099925	GO:0005737	6.58E-06	0.000973186
TCONS_00116477	GO:0042738	6.62E-06	0.000289175
TCONS_00083441	GO:0034113	6.63E-06	0.000189498
TCONS_00135735	GO:0005783	6.98E-06	0.001831886
TCONS_00116477	GO:0004879	6.98E-06	0.000289175
TCONS_00107469	GO:0005515	7.01E-06	0.001125105
TCONS_00000678	GO:0047865	7.74E-06	0.000994556
TCONS_00083441	GO:0031290	7.90E-06	0.000197479
TCONS_00059135	GO:0015293	8.27E-06	0.000901603
TCONS_00059135	GO:0015698	9.45E-06	0.000901603
TCONS_00001295	GO:0005096	1.06E-05	0.000971848
TCONS_00001295	GO:0005525	1.09E-05	0.000971848
TCONS_00059692	GO:0005515	1.12E-05	0.001149501
TCONS_00180450	GO:0043271	1.17E-05	0.002149727
TCONS_00000678	GO:0009790	1.19E-05	0.001016161
TCONS_00135735	GO:0005096	1.33E-05	0.001831886
TCONS_00066324	GO:0005654	1.39E-05	0.001435459
TCONS_00180450	GO:0015293	1.54E-05	0.002149727
TCONS_00099925	GO:0009615	1.64E-05	0.001614744
TCONS_00152371	GO:0044822	1.67E-05	0.002576453
TCONS_00135735	GO:0005743	1.71E-05	0.001831886
TCONS_00152371	GO:0005634	1.99E-05	0.002576453
TCONS_00053949	GO:0000166	2.47E-05	0.006845202
TCONS_00059135	GO:0005328	2.53E-05	0.000901603
TCONS_00180450	GO:0015698	3.07E-05	0.002149727
TCONS_00059692	GO:0005743	3.16E-05	0.002432915
TCONS_00168834	GO:0005436	3.19E-05	0.00210741
TCONS_00180450	GO:0005328	3.22E-05	0.002149727

TCONS_00054903	GO:0003743	3.33E-05	0.00848847
TCONS_00059135	GO:0035565	3.43E-05	0.000901603
TCONS_00059135	GO:0039020	3.43E-05	0.000901603
TCONS_00059135	GO:0060677	3.43E-05	0.000901603
TCONS_00059135	GO:0061017	3.43E-05	0.000901603
TCONS_00059135	GO:0061296	3.43E-05	0.000901603
TCONS_00059135	GO:0072176	3.43E-05	0.000901603
TCONS_00059135	GO:0072179	3.43E-05	0.000901603
TCONS_00059135	GO:1900212	3.43E-05	0.000901603
TCONS_00180450	GO:0003956	3.51E-05	0.002149727
TCONS_00175552	GO:0043540	3.57E-05	0.006030915
TCONS_00089741	GO:0043540	3.61E-05	0.007001035
TCONS_00099925	GO:0035455	3.91E-05	0.002890935
TCONS_00107469	GO:0005634	4.09E-05	0.004373177
TCONS_00181075	GO:0005743	4.39E-05	0.004287948
TCONS_00001295	GO:0015031	4.44E-05	0.002962467
TCONS_00181075	GO:0044822	4.59E-05	0.004287948
TCONS_00116477	GO:0048855	4.82E-05	0.001663001
TCONS_00168834	GO:0015321	4.90E-05	0.00210741
TCONS_00168834	GO:0035435	4.90E-05	0.00210741
TCONS_00168834	GO:0044341	4.90E-05	0.00210741
TCONS_00180450	GO:0006638	5.12E-05	0.002149727
TCONS_00111709	GO:0005436	5.15E-05	0.002910784
TCONS_00181075	GO:0005634	5.34E-05	0.004287948
TCONS_00001295	GO:0000166	5.53E-05	0.002962467
TCONS_00000679	GO:0001726	5.58E-05	0.009430322
TCONS_00059135	GO:0005496	5.64E-05	0.001095082
TCONS_00111709	GO:0007613	5.88E-05	0.002910784
TCONS_00008583	GO:0043540	5.91E-05	0.007290876
TCONS_00175552	GO:0004331	5.92E-05	0.006030915
TCONS_00028510	GO:0005436	5.98E-05	0.004024213
TCONS_00089741	GO:0004331	5.98E-05	0.007001035
TCONS_00059135	GO:0042663	6.06E-05	0.001095082
TCONS_00059135	GO:0060261	6.06E-05	0.001095082
TCONS_00059135	GO:0072095	6.06E-05	0.001095082
TCONS_00059135	GO:0072181	6.06E-05	0.001095082
TCONS_00175552	GO:0043116	6.44E-05	0.006030915
TCONS_00181075	GO:0005515	6.47E-05	0.004287948
TCONS_00083441	GO:0000268	6.70E-05	0.001340722
TCONS_00083441	GO:0005052	6.70E-05	0.001340722
TCONS_00089751	GO:0043540	6.73E-05	0.009519558
TCONS_00059692	GO:0005654	6.74E-05	0.004152042
TCONS_00000678	GO:0030216	6.77E-05	0.004352369
TCONS_00059135	GO:0007605	6.87E-05	0.001167803
TCONS_00107469	GO:0005737	7.27E-05	0.005836439
TCONS_00008583	GO:1903025	7.28E-05	0.007290876
TCONS_00168834	GO:0006817	7.28E-05	0.002504569

TCONS_00111709	GO:0014819	7.90E-05	0.002910784
TCONS_00111709	GO:0015321	7.90E-05	0.002910784
TCONS_00111709	GO:0015368	7.90E-05	0.002910784
TCONS_00111709	GO:0035435	7.90E-05	0.002910784
TCONS_00111709	GO:0044341	7.90E-05	0.002910784
TCONS_00083441	GO:0016925	7.94E-05	0.001444022
TCONS_00135735	GO:0005737	9.00E-05	0.00602318
TCONS_00099925	GO:0051092	9.14E-05	0.005408064
TCONS_00028510	GO:0015321	9.15E-05	0.004024213
TCONS_00028510	GO:0035435	9.15E-05	0.004024213
TCONS_00028510	GO:0044341	9.15E-05	0.004024213
TCONS_00180450	GO:0035565	9.23E-05	0.002149727
TCONS_00180450	GO:0039020	9.23E-05	0.002149727
TCONS_00180450	GO:0060677	9.23E-05	0.002149727
TCONS_00180450	GO:0061017	9.23E-05	0.002149727
TCONS_00180450	GO:0061296	9.23E-05	0.002149727
TCONS_00180450	GO:0072176	9.23E-05	0.002149727
TCONS_00180450	GO:0072179	9.23E-05	0.002149727
TCONS_00180450	GO:1900212	9.23E-05	0.002149727
TCONS_00008583	GO:0004331	9.76E-05	0.007290876
TCONS_00059135	GO:0001714	0.000100158	0.001315716
TCONS_00059135	GO:0005332	0.000100158	0.001315716
TCONS_00059135	GO:0048793	0.000100158	0.001315716
TCONS_00059135	GO:0072164	0.000100158	0.001315716
TCONS_00059135	GO:0072177	0.000100158	0.001315716
TCONS_00066324	GO:0048512	0.000103442	0.005507589
TCONS_00168834	GO:0030643	0.000104983	0.003009514
TCONS_00111709	GO:0006851	0.000105571	0.003016117
TCONS_00066324	GO:0030332	0.000106427	0.005507589
TCONS_00059135	GO:0006836	0.000107712	0.00135342
TCONS_00089751	GO:0004331	0.000111044	0.009519558
TCONS_00107469	GO:0006911	0.000112503	0.007222667
TCONS_00108000	GO:0031369	0.000113282	0.013423972
TCONS_00111709	GO:0005432	0.000116904	0.003016117
TCONS_00111709	GO:0006817	0.000116904	0.003016117
TCONS_00135735	GO:0005747	0.000120229	0.00602318
TCONS_00099739	GO:0090214	0.000129357	0.024836528
TCONS_00066898	GO:0005158	0.000132983	0.01934966
TCONS_00135735	GO:0044822	0.000133182	0.00602318
TCONS_00066898	GO:0005515	0.000133908	0.01934966
TCONS_00028510	GO:0006817	0.000135226	0.004759956
TCONS_00181075	GO:0048009	0.000136275	0.007222567
TCONS_00107469	GO:0006457	0.000140162	0.007498651
TCONS_00059692	GO:0005730	0.000147099	0.007340729
TCONS_00175552	GO:0050429	0.00014788	0.008310859
TCONS_00175552	GO:0052548	0.00014788	0.008310859
TCONS_00180450	GO:0006836	0.000148561	0.00277433

TCONS_00089741	GO:0050429	0.000149178	0.008726933
TCONS_00089741	GO:0052548	0.000149178	0.008726933
TCONS_00099925	GO:0048512	0.000150069	0.00658953
TCONS_00135735	GO:0005793	0.000150504	0.00602318
TCONS_00099925	GO:0040023	0.000155833	0.00658953
TCONS_00135735	GO:0009615	0.000155971	0.00602318
TCONS_00059135	GO:0001826	0.000156853	0.001813225
TCONS_00059135	GO:0015812	0.000156853	0.001813225
TCONS_00180450	GO:0042663	0.000161694	0.00277433
TCONS_00180450	GO:0060261	0.000161694	0.00277433
TCONS_00180450	GO:0072095	0.000161694	0.00277433
TCONS_00180450	GO:0072181	0.000161694	0.00277433
TCONS_00059135	GO:0043271	0.00016356	0.00181803
TCONS_00040392	GO:0034113	0.000165226	0.015489765
TCONS_00059692	GO:0048026	0.000166835	0.007340729
TCONS_00111709	GO:0030643	0.000167966	0.003939576
TCONS_00152371	GO:0005737	0.00016914	0.014602434
TCONS_00180450	GO:0007605	0.000174174	0.002788702
TCONS_00135735	GO:0043209	0.000175492	0.00602318
TCONS_00180450	GO:0044212	0.00017964	0.002788702
TCONS_00019890	GO:0042641	0.000180414	0.029253812
TCONS_00135735	GO:0005515	0.000187055	0.00602318
TCONS_00181075	GO:0006911	0.000188588	0.008329296
TCONS_00111709	GO:0005778	0.000191072	0.004108049
TCONS_00008583	GO:0030890	0.000191963	0.008292375
TCONS_00028510	GO:0030643	0.000194065	0.005692574
TCONS_00111084	GO:0034968	0.000197013	0.01941794
TCONS_00168834	GO:0042301	0.000202575	0.004977552
TCONS_00175552	GO:0042592	0.0002033	0.009521238
TCONS_00019890	GO:0090214	0.000209705	0.029253812
TCONS_00111084	GO:0005158	0.000212814	0.01941794
TCONS_00116477	GO:0033554	0.000218511	0.006461681
TCONS_00053949	GO:0055007	0.000219382	0.020583479
TCONS_00008583	GO:0050429	0.000222117	0.008292375
TCONS_00008583	GO:0052548	0.000222117	0.008292375
TCONS_00111084	GO:0004004	0.000222343	0.01941794
TCONS_00059135	GO:0090214	0.000226757	0.002427137
TCONS_00040392	GO:0031012	0.000228357	0.015489765
TCONS_00107469	GO:0048026	0.000233934	0.010727556
TCONS_00180450	GO:0022408	0.000236179	0.003197557
TCONS_00089035	GO:0050429	0.000236843	0.026407949
TCONS_00089035	GO:0052548	0.000236843	0.026407949
TCONS_00000678	GO:0006544	0.000242014	0.012439512
TCONS_00054903	GO:0097155	0.000246121	0.009215942
TCONS_00054903	GO:0097156	0.000246121	0.009215942
TCONS_00054903	GO:0097161	0.000246121	0.009215942
TCONS_00054903	GO:2001108	0.000246121	0.009215942

TCONS_00089751	GO:0002071	0.000246621	0.009519558
TCONS_00089751	GO:0050429	0.000246621	0.009519558
TCONS_00089751	GO:0052548	0.000246621	0.009519558
TCONS_00000679	GO:0000166	0.000247948	0.020951566
TCONS_00053949	GO:0060742	0.000251977	0.020583479
TCONS_00180450	GO:0001714	0.000264828	0.003197557
TCONS_00180450	GO:0005332	0.000264828	0.003197557
TCONS_00180450	GO:0048793	0.000264828	0.003197557
TCONS_00180450	GO:0072164	0.000264828	0.003197557
TCONS_00180450	GO:0072177	0.000264828	0.003197557
TCONS_00001295	GO:0005634	0.000266468	0.011902259
TCONS_00066898	GO:0044822	0.000267062	0.023785608
TCONS_00054903	GO:0004004	0.000270475	0.009215942
TCONS_00111709	GO:0008385	0.000270541	0.005369203
TCONS_00040392	GO:0010484	0.000285132	0.015489765
TCONS_00175552	GO:0003873	0.00028613	0.011486083
TCONS_00089741	GO:0003873	0.000289139	0.012372866
TCONS_00054903	GO:0005158	0.000290566	0.009215942
TCONS_00053949	GO:0000381	0.000297234	0.020583479
TCONS_00135735	GO:0005509	0.000298801	0.008746706
TCONS_00001295	GO:0005737	0.000321056	0.01229186
TCONS_00111709	GO:0042301	0.000321809	0.005930473
TCONS_00116477	GO:0006590	0.000326695	0.007513975
TCONS_00116477	GO:0016174	0.000326695	0.007513975
TCONS_00059135	GO:0022408	0.000326785	0.003328205
TCONS_00089741	GO:0005515	0.000332381	0.012372866
TCONS_00099925	GO:0090286	0.000338086	0.012509181
TCONS_00059135	GO:0048806	0.000339964	0.003328205
TCONS_00040392	GO:0043009	0.000342315	0.015489765
TCONS_00059135	GO:0030259	0.000345488	0.003328205
TCONS_00107469	GO:0006357	0.000347817	0.013956164
TCONS_00033508	GO:0019882	0.000352976	0.038668629
TCONS_00168834	GO:0031402	0.000360441	0.007749485
TCONS_00108000	GO:0044822	0.000360699	0.022299178
TCONS_00175552	GO:0004435	0.000364143	0.012246387
TCONS_00054903	GO:0061001	0.000370921	0.009215942
TCONS_00028510	GO:0042301	0.000370947	0.009326665
TCONS_00066324	GO:0035329	0.000373939	0.012962804
TCONS_00083441	GO:0071600	0.000380536	0.00634227
TCONS_00066324	GO:0043536	0.000387274	0.012962804
TCONS_00111709	GO:0046835	0.000390864	0.006722856
TCONS_00175552	GO:0006003	0.000392233	0.012246387
TCONS_00089741	GO:0006003	0.000396328	0.012372866
TCONS_00054903	GO:0021957	0.00039755	0.009215942
TCONS_00054903	GO:0048681	0.00039755	0.009215942
TCONS_00054903	GO:0048710	0.00039755	0.009215942
TCONS_00107469	GO:0019843	0.000401338	0.013972006

TCONS_00180450	GO:0001826	0.000411182	0.004469872
TCONS_00180450	GO:0015812	0.000411182	0.004469872
TCONS_00180450	GO:0030259	0.000411338	0.004469872
TCONS_00066898	GO:0004004	0.000411516	0.023785608
TCONS_00066898	GO:0060081	0.000411516	0.023785608
TCONS_00089741	GO:0071346	0.000423004	0.012372866
TCONS_00059135	GO:0008144	0.000424631	0.003958659
TCONS_00089751	GO:0046339	0.000428084	0.012706273
TCONS_00001295	GO:0003924	0.000428224	0.01387472
TCONS_00157353	GO:0045022	0.000432599	0.032004554
TCONS_00107469	GO:0005743	0.000435265	0.013972006
TCONS_00066324	GO:0004843	0.000438356	0.012962804
TCONS_00099925	GO:0005634	0.00043938	0.014450736
TCONS_00157353	GO:0030897	0.000442739	0.032004554
TCONS_00180450	GO:0030111	0.00045729	0.004808918
TCONS_00028509	GO:0008499	0.000457686	0.038614573
TCONS_00040392	GO:0070062	0.000457847	0.01657407
TCONS_00181075	GO:0005096	0.000462402	0.016075493
TCONS_00008583	GO:0003873	0.00046503	0.014880969
TCONS_00099739	GO:0043484	0.000482503	0.025353553
TCONS_00181075	GO:0005747	0.000485298	0.016075493
TCONS_00111084	GO:0008499	0.000496344	0.0242726
TCONS_00180450	GO:0090214	0.000501076	0.005104707
TCONS_00099925	GO:0045071	0.000501437	0.014842525
TCONS_00019890	GO:0030506	0.000505397	0.032386812
TCONS_00054903	GO:0046854	0.00051387	0.010919744
TCONS_00089751	GO:0001843	0.000514203	0.012706273
TCONS_00001295	GO:0052548	0.000514441	0.01387472
TCONS_00116477	GO:0005954	0.000515783	0.01067671
TCONS_00001295	GO:2001214	0.000517713	0.01387472
TCONS_00089751	GO:0003873	0.000526685	0.012706273
TCONS_00175552	GO:0045022	0.000527083	0.014811023
TCONS_00019890	GO:0086080	0.000527973	0.032386812
TCONS_00059692	GO:0005737	0.000528075	0.020330891
TCONS_00083441	GO:0035088	0.000533008	0.007106773
TCONS_00083441	GO:0071599	0.000533008	0.007106773
TCONS_00083441	GO:0072513	0.000533008	0.007106773
TCONS_00135735	GO:0008137	0.000533847	0.014324906
TCONS_00089741	GO:0031012	0.000540727	0.013611827
TCONS_00059135	GO:0003700	0.00054242	0.004898727
TCONS_00108000	GO:0042835	0.000553191	0.022299178
TCONS_00099925	GO:0034993	0.000558573	0.015030695
TCONS_00111709	GO:0031402	0.00056855	0.008896132
TCONS_00028509	GO:0009312	0.000573625	0.038614573
TCONS_00028509	GO:0033391	0.000573625	0.038614573
TCONS_00111709	GO:0007612	0.000586179	0.008896132
TCONS_00162688	GO:0008499	0.00058831	0.043957575

TCONS_00000679	GO:0003779	0.000589008	0.033180806
TCONS_00108000	GO:0005654	0.000592091	0.022299178
TCONS_00059135	GO:0030111	0.000595753	0.005083844
TCONS_00107469	GO:0001702	0.000596842	0.017416949
TCONS_00059135	GO:0030506	0.000598099	0.005083844
TCONS_00135735	GO:0045333	0.000604421	0.014971044
TCONS_00175552	GO:0071773	0.000608418	0.015417212
TCONS_00083441	GO:0016337	0.000609275	0.007313752
TCONS_00180450	GO:0042312	0.000611172	0.006037643
TCONS_00108000	GO:0046854	0.000631244	0.022299178
TCONS_00008583	GO:0006003	0.00063513	0.017783647
TCONS_00019890	GO:0045162	0.000635616	0.032386812
TCONS_00089741	GO:0060346	0.000639305	0.013611827
TCONS_00089741	GO:0043209	0.000639872	0.013611827
TCONS_00033508	GO:0030866	0.000641156	0.038668629
TCONS_00028510	GO:0031402	0.000653848	0.014384666
TCONS_00059692	GO:0006457	0.000654248	0.02039883
TCONS_00066898	GO:0005634	0.000659488	0.031765331
TCONS_00099739	GO:0005765	0.000660403	0.025353553
TCONS_00089035	GO:0043540	0.000661294	0.042992185
TCONS_00059692	GO:0030529	0.0006623	0.02039883
TCONS_00107469	GO:0046854	0.000678043	0.018137655
TCONS_00175552	GO:1901522	0.000690191	0.015417212
TCONS_00089751	GO:0005524	0.000692413	0.013869127
TCONS_00019890	GO:0023051	0.000696491	0.032386812
TCONS_00083441	GO:0031267	0.000703484	0.007313752
TCONS_00111084	GO:0016192	0.0007046	0.0242726
TCONS_00054903	GO:0006413	0.000711929	0.013774444
TCONS_00175552	GO:0006886	0.000713252	0.015417212
TCONS_00089751	GO:0006003	0.000718608	0.013869127
TCONS_00000678	GO:0008544	0.000725245	0.030974628
TCONS_00083441	GO:0007501	0.000727163	0.007313752
TCONS_00083441	GO:0014706	0.000727163	0.007313752
TCONS_00083441	GO:0010212	0.000731375	0.007313752
TCONS_00108000	GO:0043484	0.000737987	0.022299178
TCONS_00053949	GO:0003729	0.000741665	0.033041482
TCONS_00001295	GO:0031532	0.000743466	0.018113524
TCONS_00107469	GO:0030029	0.000749537	0.0185078
TCONS_00108000	GO:0031492	0.000752715	0.022299178
TCONS_00054903	GO:0033135	0.000756244	0.013774444
TCONS_00162688	GO:0009312	0.000759518	0.043957575
TCONS_00162688	GO:0033391	0.000759518	0.043957575
TCONS_00152371	GO:0043484	0.000761582	0.039091718
TCONS_00059135	GO:0060993	0.000774584	0.006395853
TCONS_00008583	GO:0032259	0.00078712	0.019590545
TCONS_00053949	GO:0022904	0.000787937	0.033041482
TCONS_00033508	GO:0005515	0.000795235	0.038668629

TCONS_00152371	GO:0005096	0.000801971	0.039091718
TCONS_00059692	GO:0005739	0.000808322	0.02252472
TCONS_00157353	GO:0043951	0.000823681	0.032004554
TCONS_00116477	GO:0035694	0.000827503	0.014274429
TCONS_00116477	GO:0035695	0.000827503	0.014274429
TCONS_00019890	GO:0005634	0.000839775	0.033471033
TCONS_00111709	GO:0004004	0.000841821	0.01149231
TCONS_00000678	GO:2000393	0.000843667	0.030974628
TCONS_00111709	GO:0051560	0.000846333	0.01149231
TCONS_00181075	GO:0048026	0.000848509	0.022644513
TCONS_00053949	GO:0006355	0.000856626	0.033041482
TCONS_00059135	GO:0033192	0.000868641	0.006973259
TCONS_00180450	GO:0048806	0.00087605	0.00839977
TCONS_00059692	GO:0005681	0.000877586	0.02252472
TCONS_00099925	GO:0043153	0.000882926	0.021778851
TCONS_00054903	GO:0042802	0.000884695	0.015039819
TCONS_00135735	GO:0006911	0.000887852	0.020420589
TCONS_00040392	GO:0033593	0.000918623	0.023752967
TCONS_00040392	GO:0070200	0.000918623	0.023752967
TCONS_00099739	GO:0005681	0.000919929	0.025353553
TCONS_00033508	GO:0033141	0.00092003	0.038668629
TCONS_00175552	GO:0001710	0.000921219	0.015461895
TCONS_00175552	GO:0005515	0.000930393	0.015461895
TCONS_00001295	GO:0000502	0.000931704	0.020808055
TCONS_00180450	GO:0043401	0.000940067	0.008694451
TCONS_00168834	GO:0061458	0.000950637	0.018167724
TCONS_00111084	GO:0048771	0.000952949	0.0242726
TCONS_00180450	GO:0006471	0.000960123	0.008694451
TCONS_00059135	GO:0004883	0.000961092	0.007011245
TCONS_00059135	GO:0038051	0.000961092	0.007011245
TCONS_00059135	GO:0043402	0.000961092	0.007011245
TCONS_00083441	GO:0048752	0.000969642	0.008814924
TCONS_00083441	GO:0048856	0.000969642	0.008814924
TCONS_00059135	GO:0006366	0.000970414	0.007011245
TCONS_00000678	GO:2001214	0.000972415	0.031238846
TCONS_00175552	GO:0043394	0.000973875	0.015461895
TCONS_00099739	GO:0042116	0.000976913	0.025353553
TCONS_00108000	GO:0003729	0.000997881	0.024774593
TCONS_00089035	GO:0004331	0.001002613	0.042992185
TCONS_00099739	GO:0048771	0.001009418	0.025353553
TCONS_00111709	GO:2000147	0.001013894	0.012344873
TCONS_00066324	GO:0005737	0.001014269	0.026244209
TCONS_00157353	GO:0048771	0.00102327	0.032004554
TCONS_00099739	GO:0005515	0.001024932	0.025353553
TCONS_00111709	GO:0015095	0.001028423	0.012344873
TCONS_00019890	GO:0097225	0.001037683	0.036189196
TCONS_00111709	GO:0003743	0.001052664	0.012344873

TCONS_00053949	GO:1904417	0.001063494	0.033041482
TCONS_00168834	GO:0008378	0.001063727	0.018296099
TCONS_00181075	GO:0006367	0.001066665	0.022644513
TCONS_00053949	GO:0002021	0.00107355	0.033041482
TCONS_00054903	GO:0048771	0.00107746	0.016339756
TCONS_00107469	GO:0030036	0.001082345	0.022894769
TCONS_00116477	GO:0006805	0.00108519	0.016548874
TCONS_00054903	GO:0016307	0.001089317	0.016339756
TCONS_00107469	GO:0005747	0.001098935	0.022894769
TCONS_00181075	GO:0003714	0.001099005	0.022644513
TCONS_00152371	GO:0001726	0.001112583	0.039091718
TCONS_00135735	GO:0002526	0.001113834	0.023910314
TCONS_00175552	GO:0033141	0.00111618	0.015461895
TCONS_00180450	GO:0005496	0.001118135	0.009851675
TCONS_00116477	GO:0004601	0.001119248	0.016548874
TCONS_00152371	GO:0005515	0.001120272	0.039091718
TCONS_00059135	GO:0001077	0.001122118	0.007909563
TCONS_00089741	GO:0031056	0.001123535	0.020992453
TCONS_00175552	GO:0071672	0.001131197	0.015461895
TCONS_00099925	GO:0005521	0.001135339	0.022372565
TCONS_00107469	GO:0003714	0.001141172	0.022894769
TCONS_00181075	GO:0060612	0.001144746	0.022644513
TCONS_00059692	GO:0030855	0.001151809	0.027035146
TCONS_00181075	GO:0008137	0.001152873	0.022644513
TCONS_00108000	GO:0048771	0.00115325	0.024774593
TCONS_00033508	GO:0006886	0.001154414	0.038668629
TCONS_00001295	GO:0005801	0.001158821	0.022424426
TCONS_00089751	GO:0003779	0.001161084	0.020371747
TCONS_00175552	GO:0097178	0.001167739	0.015461895
TCONS_00033508	GO:0043219	0.0011699	0.038668629
TCONS_00001295	GO:0001817	0.001171425	0.022424426
TCONS_00181075	GO:0015629	0.001196314	0.022644513
TCONS_00180450	GO:0051454	0.001217753	0.010179162
TCONS_00180450	GO:0060081	0.001217753	0.010179162
TCONS_00107469	GO:0005730	0.001218505	0.023008245
TCONS_00033508	GO:0042127	0.001220342	0.038668629
TCONS_00000678	GO:0032968	0.001223026	0.034924198
TCONS_00066898	GO:0033180	0.001235709	0.042234729
TCONS_00066898	GO:0043951	0.001235709	0.042234729
TCONS_00111709	GO:0015693	0.00123992	0.013908665
TCONS_00157353	GO:0070695	0.001240653	0.032004554
TCONS_00157353	GO:1903206	0.001240653	0.032004554
TCONS_00033508	GO:0019722	0.00124573	0.038668629
TCONS_00108000	GO:0000790	0.00124596	0.024774593
TCONS_00083441	GO:0048665	0.001267381	0.011020709
TCONS_00108000	GO:0006376	0.001267852	0.024774593
TCONS_00066324	GO:0005515	0.001280751	0.028992699

TCONS_00175552	GO:0002051	0.001289285	0.015461895
TCONS_00059692	GO:0006778	0.001291615	0.027035146
TCONS_00089741	GO:0000435	0.001300135	0.020992453
TCONS_00089741	GO:2001038	0.001300135	0.020992453
TCONS_00152371	GO:0008203	0.001312724	0.039091718
TCONS_00059135	GO:0031290	0.001314695	0.009046354
TCONS_00059692	GO:0008429	0.001316647	0.027035146
TCONS_00099739	GO:0005634	0.001324611	0.025353553
TCONS_00099925	GO:0035212	0.001343805	0.022372565
TCONS_00099925	GO:1990244	0.001343805	0.022372565
TCONS_00099925	GO:1990245	0.001343805	0.022372565
TCONS_00108000	GO:0005681	0.001358944	0.024774593
TCONS_00099925	GO:0033151	0.001360494	0.022372565
TCONS_00099925	GO:0038028	0.001360494	0.022372565
TCONS_00089035	GO:0005634	0.001374455	0.042992185
TCONS_00180450	GO:0010742	0.001377253	0.011140659
TCONS_00099739	GO:0086080	0.001377363	0.025353553
TCONS_00083441	GO:0005681	0.001378027	0.011483558
TCONS_00054903	GO:0000981	0.001388073	0.019664362
TCONS_00053949	GO:0005515	0.001388632	0.037656911
TCONS_00059135	GO:0019276	0.001391558	0.009140008
TCONS_00059135	GO:0051428	0.001391558	0.009140008
TCONS_00033508	GO:0090004	0.001395275	0.038668629
TCONS_00066324	GO:0003365	0.001400613	0.028992699
TCONS_00180450	GO:0031012	0.001401126	0.011140659
TCONS_00066898	GO:0048771	0.00140281	0.042234729
TCONS_00175552	GO:0006000	0.001416773	0.015461895
TCONS_00175552	GO:0043237	0.001416773	0.015461895
TCONS_00175552	GO:0070695	0.001416773	0.015461895
TCONS_00107469	GO:0034663	0.00142387	0.025392355
TCONS_00089741	GO:0006000	0.001431033	0.020992453
TCONS_00168834	GO:0008499	0.001438504	0.019770412
TCONS_00135735	GO:0043547	0.001454074	0.029237396
TCONS_00111084	GO:0035264	0.001454852	0.0242726
TCONS_00099739	GO:0019227	0.001456872	0.025353553
TCONS_00001295	GO:0043547	0.001458933	0.026066266
TCONS_00059135	GO:0060429	0.001460682	0.00931721
TCONS_00059135	GO:0046854	0.001483016	0.00931721
TCONS_00152371	GO:0016805	0.001495723	0.039091718
TCONS_00157353	GO:0070534	0.001496474	0.032004554
TCONS_00099739	GO:0043983	0.001504302	0.025353553
TCONS_00066898	GO:0030117	0.001508147	0.042234729
TCONS_00152371	GO:0003729	0.001509333	0.039091718
TCONS_00089741	GO:0004435	0.001525093	0.020992453
TCONS_00089741	GO:0016409	0.001525093	0.020992453
TCONS_00181075	GO:0016805	0.001531063	0.024465267
TCONS_00135735	GO:0060612	0.001543589	0.029237396

TCONS_00028509	GO:0045056	0.00155129	0.038614573
TCONS_00181075	GO:0046854	0.001556799	0.024465267
TCONS_00116477	GO:0042403	0.001557495	0.021493428
TCONS_00033508	GO:0045056	0.001565883	0.038668629
TCONS_00181075	GO:0005669	0.00156947	0.024465267
TCONS_00108000	GO:0005515	0.001598512	0.026121407
TCONS_00053949	GO:0010447	0.001604573	0.037656911
TCONS_00066324	GO:0016579	0.001614613	0.030380628
TCONS_00099739	GO:0045162	0.001619271	0.025353553
TCONS_00111084	GO:0045056	0.001621358	0.0242726
TCONS_00107469	GO:0006778	0.001626404	0.026875688
TCONS_00040392	GO:0086080	0.001636391	0.031620092
TCONS_00059135	GO:0043401	0.001637575	0.010069344
TCONS_00180450	GO:0048240	0.001638404	0.012717133
TCONS_00099925	GO:0090292	0.001638555	0.025526969
TCONS_00028510	GO:0046835	0.001640422	0.032079357
TCONS_00175552	GO:0048846	0.001642674	0.015461895
TCONS_00000679	GO:0000381	0.001648428	0.042433189
TCONS_00083441	GO:0052695	0.001651664	0.012324837
TCONS_00108000	GO:0043044	0.001653254	0.026121407
TCONS_00059692	GO:0033120	0.001655203	0.031862664
TCONS_00066898	GO:0001726	0.001662045	0.042234729
TCONS_00083441	GO:0001656	0.001663853	0.012324837
TCONS_00083441	GO:0031492	0.001663853	0.012324837
TCONS_00000678	GO:0060020	0.001707392	0.036927041
TCONS_00000678	GO:1900028	0.001707392	0.036927041
TCONS_00111084	GO:0005515	0.001708678	0.0242726
TCONS_00099739	GO:0044822	0.001716647	0.025353553
TCONS_00168834	GO:0060480	0.001724164	0.019770412
TCONS_00168834	GO:0070254	0.001724164	0.019770412
TCONS_00168834	GO:1903896	0.001724164	0.019770412
TCONS_00168834	GO:1903899	0.001724164	0.019770412

Supplementary 2

lncRNA	Term_ID	P_value	Q_value
TCONS_00135735	path:ko04141	6.08E-09	1.70E-07
TCONS_00107469	path:ko04141	5.09E-06	0.00014259
TCONS_00107469	path:ko03060	1.51E-05	0.000210961
TCONS_00059692	path:ko03050	4.42E-05	0.000826449
TCONS_00099925	path:ko04141	5.31E-05	0.000796437
TCONS_00059692	path:ko04068	6.04E-05	0.000826449
TCONS_00135735	path:ko03050	8.08E-05	0.000917869
TCONS_00135735	path:ko00190	9.83E-05	0.000917869
TCONS_00059692	path:ko03060	0.000122301	0.000826449
TCONS_00001295	path:ko03040	0.000123513	0.002134295
TCONS_00059692	path:ko04722	0.000124085	0.000826449
TCONS_00059692	path:ko03040	0.000129133	0.000826449
TCONS_00135735	path:ko03060	0.00013799	0.000965933
TCONS_00152371	path:ko00190	0.000142662	0.003851864
TCONS_00066898	path:ko04722	0.000182799	0.003858247
TCONS_00135735	path:ko04722	0.000187154	0.001048064
TCONS_00107469	path:ko04068	0.000221813	0.001913475
TCONS_00001295	path:ko03050	0.000237144	0.002134295
TCONS_00066898	path:ko04670	0.00024379	0.003858247
TCONS_00152371	path:ko03040	0.000301974	0.004076654
TCONS_00181075	path:ko00190	0.000326565	0.004797554
TCONS_00107469	path:ko00190	0.000331477	0.001913475
TCONS_00107469	path:ko03040	0.000368639	0.001913475
TCONS_00107469	path:ko03050	0.00041003	0.001913475
TCONS_00000678	path:ko04919	0.000412981	0.007433666
TCONS_00181075	path:ko04919	0.000417179	0.004797554
TCONS_00066898	path:ko04068	0.000465869	0.003858247
TCONS_00066898	path:ko04210	0.000482281	0.003858247
TCONS_00028509	path:ko00603	0.000524472	0.015734156
TCONS_00089751	path:ko03050	0.000622963	0.005606664
TCONS_00089741	path:ko04720	0.000632893	0.010556598
TCONS_00107469	path:ko00010	0.000666492	0.002665967
TCONS_00089741	path:ko04922	0.000703773	0.010556598
TCONS_00001295	path:ko00190	0.000739831	0.004438983
TCONS_00181075	path:ko04722	0.000757495	0.005807461
TCONS_00116477	path:ko00360	0.00078743	0.006299442
TCONS_00157353	path:ko00750	0.000895709	0.013435642
TCONS_00019890	path:ko04670	0.000924928	0.016648704
TCONS_00083441	path:ko00140	0.000959328	0.009963367
TCONS_00111084	path:ko04670	0.001052477	0.016482876
TCONS_00099925	path:ko03050	0.001130019	0.008475142
TCONS_00089741	path:ko00562	0.001138397	0.011383969
TCONS_00181075	path:ko03040	0.001175842	0.006761093
TCONS_00175552	path:ko00562	0.00117592	0.01963179
TCONS_00108000	path:ko03040	0.001234525	0.011115262

TCONS_00108000	path:ko00190	0.00135029	0.011115262
TCONS_00066898	path:ko04919	0.001393304	0.008917148
TCONS_00107469	path:ko04722	0.001476476	0.005167667
TCONS_00111709	path:ko04022	0.001506938	0.026661838
TCONS_00083441	path:ko00601	0.001532826	0.009963367
TCONS_00089741	path:ko04150	0.001537024	0.011527683
TCONS_00108000	path:ko04150	0.001587895	0.011115262
TCONS_00099739	path:ko03015	0.001604306	0.013679579
TCONS_00175552	path:ko00020	0.001675195	0.01963179
TCONS_00152371	path:ko04068	0.001739798	0.015658184
TCONS_00040392	path:ko04514	0.00175543	0.026331449
TCONS_00053949	path:ko04670	0.001916783	0.025738377
TCONS_00008583	path:ko00440	0.00193722	0.032923679
TCONS_00135735	path:ko04071	0.001943233	0.00906842
TCONS_00066898	path:ko04142	0.001953465	0.010368977
TCONS_00180450	path:ko02010	0.001964778	0.044448631
TCONS_00175552	path:ko04728	0.002103406	0.01963179
TCONS_00089035	path:ko04111	0.002225562	0.023468831
TCONS_00059692	path:ko00190	0.0022401	0.011947198
TCONS_00001295	path:ko04142	0.002243893	0.01009752
TCONS_00066898	path:ko04664	0.002268214	0.010368977
TCONS_00111084	path:ko04910	0.002273655	0.016482876
TCONS_00181075	path:ko00620	0.002321575	0.010679245
TCONS_00111709	path:ko04071	0.002359906	0.026661838
TCONS_00111084	path:ko04068	0.002378883	0.016482876
TCONS_00028509	path:ko00562	0.002415609	0.036234134
TCONS_00099739	path:ko04670	0.002487196	0.013679579
TCONS_00111084	path:ko04150	0.002524431	0.016482876
TCONS_00059135	path:ko00830	0.002677087	0.037479222
TCONS_00053949	path:ko04919	0.002700781	0.025738377
TCONS_00116477	path:ko00563	0.002808589	0.011234355
TCONS_00089741	path:ko04724	0.002818202	0.016768246
TCONS_00111709	path:ko04014	0.002856625	0.026661838
TCONS_00089035	path:ko04670	0.002933604	0.023468831
TCONS_00001295	path:ko04011	0.002939474	0.010582108
TCONS_00152371	path:ko04722	0.002959714	0.018282165
TCONS_00111084	path:ko04066	0.003006026	0.016482876
TCONS_00111084	path:ko04014	0.003317887	0.016482876
TCONS_00089741	path:ko04662	0.003353649	0.016768246
TCONS_00181075	path:ko04141	0.00343243	0.013157648
TCONS_00152371	path:ko03050	0.003491312	0.018282165
TCONS_00040392	path:ko04662	0.003644816	0.027336123
TCONS_00111084	path:ko04722	0.003722733	0.016482876
TCONS_00107469	path:ko04621	0.003775219	0.011745127
TCONS_00053949	path:ko04142	0.003811702	0.025738377
TCONS_00111084	path:ko04210	0.003878324	0.016482876
TCONS_00053949	path:ko04530	0.00411814	0.025738377

TCONS_00152371	path:ko03013	0.004148262	0.018282165
TCONS_00108000	path:ko04068	0.004165251	0.020896786
TCONS_00066898	path:ko03040	0.00418392	0.016735681
TCONS_00111709	path:ko04664	0.004299252	0.02983066
TCONS_00059692	path:ko04141	0.004460006	0.018906271
TCONS_00001295	path:ko04145	0.004463293	0.01338988
TCONS_00111084	path:ko04919	0.004468984	0.016882829
TCONS_00028509	path:ko00563	0.004471245	0.039109544
TCONS_00008583	path:ko04922	0.004605765	0.032923679
TCONS_00089035	path:ko04142	0.004645041	0.02477355
TCONS_00089741	path:ko04213	0.004654452	0.017620092
TCONS_00000679	path:ko03015	0.004692561	0.037290544
TCONS_00089741	path:ko04919	0.004698691	0.017620092
TCONS_00033508	path:ko00563	0.004714282	0.031091362
TCONS_00059692	path:ko03013	0.004726568	0.018906271
TCONS_00135735	path:ko03040	0.004761593	0.017619185
TCONS_00181075	path:ko04310	0.004851262	0.01593986
TCONS_00108000	path:ko04620	0.004975425	0.020896786
TCONS_00152371	path:ko04150	0.004980423	0.018282165
TCONS_00028510	path:ko04750	0.005010231	0.0472033
TCONS_00135735	path:ko00020	0.005034053	0.017619185
TCONS_00175552	path:ko04724	0.005365837	0.037560858
TCONS_00054903	path:ko00562	0.005409663	0.049956277
TCONS_00152371	path:ko03015	0.005416938	0.018282165
TCONS_00111084	path:ko00310	0.005495712	0.018685421
TCONS_00008583	path:ko00310	0.005514824	0.032923679
TCONS_00001295	path:ko00020	0.005574174	0.014333589
TCONS_00059692	path:ko04919	0.005656209	0.020110966
TCONS_00066324	path:ko00440	0.005698741	0.044355285
TCONS_00053949	path:ko04510	0.005739792	0.028698961
TCONS_00135735	path:ko04730	0.005949121	0.018508376
TCONS_00019890	path:ko04727	0.005973279	0.023578991
TCONS_00033508	path:ko00562	0.005986717	0.031091362
TCONS_00111709	path:ko04915	0.006036923	0.02983066
TCONS_00040392	path:ko00440	0.006038877	0.029537008
TCONS_00059135	path:ko02010	0.006170881	0.042952503
TCONS_00019890	path:ko04520	0.006356399	0.023578991
TCONS_00019890	path:ko00900	0.006377995	0.023578991
TCONS_00111709	path:ko04972	0.006392284	0.02983066
TCONS_00019890	path:ko04390	0.00654972	0.023578991
TCONS_00059692	path:ko04621	0.006650096	0.021280307
TCONS_00099739	path:ko04111	0.006789106	0.022506227
TCONS_00008583	path:ko04919	0.006931301	0.032923679
TCONS_00028509	path:ko00860	0.006952108	0.039109544
TCONS_00066898	path:ko03015	0.007071436	0.024512659
TCONS_00089741	path:ko04750	0.007144874	0.021567946
TCONS_00135735	path:ko04540	0.007181412	0.020107954

TCONS_00089035	path:ko04113	0.007186373	0.028745492
TCONS_00000679	path:ko04962	0.007186603	0.037290544
TCONS_00111084	path:ko04664	0.007246503	0.022398282
TCONS_00108000	path:ko04919	0.007289932	0.025514761
TCONS_00000679	path:ko04670	0.007292573	0.037290544
TCONS_00181075	path:ko04730	0.007313653	0.021026752
TCONS_00033508	path:ko04630	0.007433101	0.031091362
TCONS_00168834	path:ko00510	0.007448373	0.031895438
TCONS_00099925	path:ko04120	0.007498373	0.037491864
TCONS_00066898	path:ko04011	0.007660206	0.024512659
TCONS_00054903	path:ko04919	0.00767683	0.049956277
TCONS_00180450	path:ko04727	0.007934834	0.044448631
TCONS_00168834	path:ko04668	0.007938484	0.031895438
TCONS_00157353	path:ko00052	0.008031184	0.045002043
TCONS_00107469	path:ko04390	0.008074777	0.022609376
TCONS_00089741	path:ko04062	0.008188465	0.021567946
TCONS_00040392	path:ko00310	0.008285452	0.029537008
TCONS_00180450	path:ko04520	0.008360938	0.044448631
TCONS_00089741	path:ko04070	0.008435812	0.021567946
TCONS_00152371	path:ko04530	0.008461687	0.02538506
TCONS_00089741	path:ko04611	0.008627178	0.021567946
TCONS_00175552	path:ko00750	0.00863519	0.038639818
TCONS_00083441	path:ko04514	0.008713586	0.030432493
TCONS_00107469	path:ko03008	0.008956811	0.022799156
TCONS_00175552	path:ko01210	0.009024309	0.038639818
TCONS_00180450	path:ko04916	0.009087396	0.044448631
TCONS_00053949	path:ko04620	0.009190909	0.03548045
TCONS_00066898	path:ko04071	0.009195233	0.026713192
TCONS_00000679	path:ko04390	0.009322636	0.037290544
TCONS_00028510	path:ko03460	0.009411867	0.0472033
TCONS_00111084	path:ko04668	0.009631408	0.027288989
TCONS_00111709	path:ko00565	0.009769351	0.033123865
TCONS_00089035	path:ko00900	0.009849447	0.031518231
TCONS_00111709	path:ko04072	0.0099278	0.033123865
TCONS_00053949	path:ko04211	0.009934526	0.03548045
TCONS_00107469	path:ko00620	0.010032089	0.023408207
TCONS_00099925	path:ko00630	0.010205834	0.038271878
TCONS_00028509	path:ko04070	0.010243245	0.039109544
TCONS_00059692	path:ko04146	0.010325735	0.02650872
TCONS_00008583	path:ko00860	0.01045523	0.034947739
TCONS_00033508	path:ko00600	0.010570374	0.031091362
TCONS_00083441	path:ko00040	0.010581803	0.030432493
TCONS_00028510	path:ko04915	0.010586921	0.0472033
TCONS_00116477	path:ko00340	0.01069288	0.026811614
TCONS_00066898	path:ko04660	0.010817012	0.026713192
TCONS_00028510	path:ko04064	0.010921972	0.0472033
TCONS_00175552	path:ko04350	0.011102961	0.038639818

TCONS_00181075	path:ko03013	0.011143699	0.026011223
TCONS_00099739	path:ko04514	0.011249838	0.022506227
TCONS_00181075	path:ko04071	0.011309227	0.026011223
TCONS_00028510	path:ko04972	0.011385839	0.0472033
TCONS_00108000	path:ko03013	0.011396274	0.027045907
TCONS_00008583	path:ko04360	0.011497151	0.034947739
TCONS_00066898	path:ko04668	0.011574581	0.026713192
TCONS_00180450	path:ko04670	0.011578721	0.044448631
TCONS_00059692	path:ko04013	0.011599754	0.02650872
TCONS_00066898	path:ko04014	0.011687022	0.026713192
TCONS_00083441	path:ko00830	0.011704805	0.030432493
TCONS_00162688	path:ko00860	0.011719605	0.040915995
TCONS_00180450	path:ko00830	0.011725652	0.044448631
TCONS_00066324	path:ko04380	0.011831808	0.044355285
TCONS_00059692	path:ko04662	0.011861603	0.02650872
TCONS_00028509	path:ko04391	0.011956789	0.039109544
TCONS_00033508	path:ko04066	0.011958216	0.031091362
TCONS_00152371	path:ko04919	0.011979539	0.028628991
TCONS_00059692	path:ko03070	0.011991574	0.02650872
TCONS_00053949	path:ko03040	0.012018584	0.037081412
TCONS_00099739	path:ko04142	0.012049052	0.022506227
TCONS_00162688	path:ko00603	0.012162183	0.040915995
TCONS_00111709	path:ko04962	0.012239388	0.033123865
TCONS_00059692	path:ko04917	0.012425963	0.02650872
TCONS_00175552	path:ko04360	0.012486279	0.038639818
TCONS_00099739	path:ko00190	0.012524125	0.022506227
TCONS_00181075	path:ko04070	0.012531488	0.026202202
TCONS_00054903	path:ko04011	0.012554432	0.049956277
TCONS_00152371	path:ko04145	0.012575965	0.028628991
TCONS_00162688	path:ko00562	0.012613712	0.040915995
TCONS_00111084	path:ko04071	0.012664344	0.032853428
TCONS_00152371	path:ko00232	0.012723996	0.028628991
TCONS_00059135	path:ko04710	0.012737519	0.042952503
TCONS_00008583	path:ko04070	0.012875483	0.034947739
TCONS_00111709	path:ko03013	0.012893088	0.033123865
TCONS_00175552	path:ko04922	0.012906754	0.038639818
TCONS_00028509	path:ko04713	0.013084777	0.039109544
TCONS_00066898	path:ko04910	0.01309667	0.027182338
TCONS_00040392	path:ko04510	0.013188784	0.029537008
TCONS_00108000	path:ko04213	0.013188828	0.027045907
TCONS_00028509	path:ko04973	0.013191932	0.039109544
TCONS_00099925	path:ko04064	0.013199291	0.039597872
TCONS_00089751	path:ko04142	0.013227653	0.029000268
TCONS_00028509	path:ko04211	0.013230338	0.039109544
TCONS_00111709	path:ko00603	0.013408428	0.033123865
TCONS_00066898	path:ko04620	0.013591169	0.027182338
TCONS_00168834	path:ko04664	0.013645965	0.031895438

TCONS_00053949	path:ko04072	0.01365081	0.037081412
TCONS_00040392	path:ko04611	0.013764889	0.029537008
TCONS_00040392	path:ko04512	0.013783937	0.029537008
TCONS_00108000	path:ko04310	0.013789924	0.027045907
TCONS_00175552	path:ko04725	0.013799935	0.038639818
TCONS_00168834	path:ko04012	0.013800292	0.031895438
TCONS_00000678	path:ko04130	0.01402669	0.037853323
TCONS_00028509	path:ko04668	0.014084875	0.039109544
TCONS_00108000	path:ko04660	0.014088297	0.027045907
TCONS_00108000	path:ko04910	0.014166904	0.027045907
TCONS_00135735	path:ko00604	0.014278251	0.036307658
TCONS_00099739	path:ko00510	0.014322144	0.022506227
TCONS_00116477	path:ko04630	0.01432828	0.026811614
TCONS_00089741	path:ko00310	0.014390294	0.03320837
TCONS_00111709	path:ko04919	0.014423629	0.033123865
TCONS_00028510	path:ko04391	0.014488579	0.0472033
TCONS_00033508	path:ko00020	0.01452461	0.031469989
TCONS_00066898	path:ko04391	0.014698308	0.02752654
TCONS_00107469	path:ko04391	0.014782449	0.031839122
TCONS_00089751	path:ko04114	0.015131644	0.029000268
TCONS_00111084	path:ko00562	0.015277847	0.032853428
TCONS_00111709	path:ko04530	0.015378938	0.033123865
TCONS_00066898	path:ko04212	0.015483679	0.02752654
TCONS_00157353	path:ko04390	0.015484514	0.045002043
TCONS_00059692	path:ko03008	0.015519772	0.029880071
TCONS_00000678	path:ko00380	0.015527853	0.037853323
TCONS_00135735	path:ko04072	0.015560425	0.036307658
TCONS_00083441	path:ko04975	0.015576866	0.033749876
TCONS_00180450	path:ko04111	0.015592154	0.044448631
TCONS_00089035	path:ko04922	0.015716317	0.039842796
TCONS_00053949	path:ko04062	0.015856446	0.037081412
TCONS_00059692	path:ko00232	0.015873788	0.029880071
TCONS_00152371	path:ko04142	0.01589001	0.033002329
TCONS_00059135	path:ko04727	0.016016666	0.042952503
TCONS_00168834	path:ko04966	0.016086859	0.031895438
TCONS_00181075	path:ko04068	0.016317194	0.031274623
TCONS_00111084	path:ko03015	0.016375487	0.032853428
TCONS_00089741	path:ko04020	0.016424554	0.035195473
TCONS_00168834	path:ko04360	0.016479712	0.031895438
TCONS_00111084	path:ko04530	0.016493836	0.032853428
TCONS_00008583	path:ko04142	0.016707191	0.037993683
TCONS_00175552	path:ko01230	0.016721814	0.039269201
TCONS_00116477	path:ko00750	0.016757259	0.026811614
TCONS_00162688	path:ko04910	0.016775948	0.040915995
TCONS_00059135	path:ko04610	0.016784255	0.042952503
TCONS_00000679	path:ko00480	0.016816654	0.045130645
TCONS_00053949	path:ko04152	0.016826732	0.037081412

TCONS_00028510	path:ko04011	0.016926138	0.0472033
TCONS_00028509	path:ko04962	0.016957867	0.039109544
TCONS_00111084	path:ko04211	0.017071402	0.032853428
TCONS_00180450	path:ko00900	0.017073705	0.044448631
TCONS_00040392	path:ko04120	0.017161774	0.031632002
TCONS_00059692	path:ko04660	0.017251812	0.03021989
TCONS_00089751	path:ko04360	0.017389231	0.029000268
TCONS_00111084	path:ko04145	0.017392991	0.032853428
TCONS_00089035	path:ko00051	0.017431223	0.039842796
TCONS_00135735	path:ko00010	0.017434684	0.037551627
TCONS_00028509	path:ko04071	0.017714738	0.039109544
TCONS_00111709	path:ko04722	0.017715141	0.033189773
TCONS_00000678	path:ko00260	0.017750108	0.037853323
TCONS_00000678	path:ko04011	0.017750108	0.037853323
TCONS_00111709	path:ko04921	0.017780235	0.033189773
TCONS_00162688	path:ko04710	0.017842426	0.040915995
TCONS_00089751	path:ko04660	0.01785869	0.029000268
TCONS_00053949	path:ko04022	0.017860151	0.037081412
TCONS_00162688	path:ko04610	0.017894544	0.040915995
TCONS_00152371	path:ko04670	0.017931101	0.034581409
TCONS_00059692	path:ko04024	0.017943059	0.03021989
TCONS_00028509	path:ko04213	0.017948911	0.039109544
TCONS_00008583	path:ko00051	0.017997008	0.037993683
TCONS_00107469	path:ko04013	0.018119269	0.035491753
TCONS_00066324	path:ko03460	0.018218115	0.044355285
TCONS_00000678	path:ko00052	0.018248597	0.037853323
TCONS_00108000	path:ko00510	0.018656912	0.032649597
TCONS_00000678	path:ko04142	0.018752196	0.037853323
TCONS_00175552	path:ko00360	0.018763894	0.039269201
TCONS_00054903	path:ko04360	0.018880745	0.049956277
TCONS_00107469	path:ko03013	0.019013439	0.035491753
TCONS_00001295	path:ko04962	0.019047171	0.042856134
TCONS_00175552	path:ko04720	0.019049384	0.039269201
TCONS_00000679	path:ko03040	0.019113193	0.045130645
TCONS_00089741	path:ko04923	0.019173757	0.038034353
TCONS_00028509	path:ko04310	0.019184547	0.039109544
TCONS_00000678	path:ko00281	0.019635742	0.037853323
TCONS_00066898	path:ko04150	0.019678669	0.033143021
TCONS_00180450	path:ko04113	0.019808437	0.044448631
TCONS_00089741	path:ko04510	0.020284988	0.038034353
TCONS_00040392	path:ko03460	0.020300081	0.031632002
TCONS_00059692	path:ko04145	0.02050398	0.031430466
TCONS_00059692	path:ko04070	0.020626243	0.031430466
TCONS_00152371	path:ko04071	0.020631272	0.03713629
TCONS_00107469	path:ko00232	0.020650535	0.036138436
TCONS_00089751	path:ko04113	0.02066367	0.029000268
TCONS_00168834	path:ko00603	0.020692803	0.031895438

TCONS_00181075	path:ko04540	0.020694005	0.034608137
TCONS_00135735	path:ko04360	0.020778541	0.041557082
TCONS_00000678	path:ko03040	0.020806219	0.037853323
TCONS_00180450	path:ko04015	0.020919413	0.044448631
TCONS_00000678	path:ko03015	0.021029624	0.037853323
TCONS_00175552	path:ko04320	0.021057216	0.039269201
TCONS_00181075	path:ko04011	0.021065822	0.034608137
TCONS_00059135	path:ko00480	0.021117413	0.042952503
TCONS_00111709	path:ko04910	0.021221649	0.035108043
TCONS_00111709	path:ko04212	0.021315598	0.035108043
TCONS_00108000	path:ko04722	0.021393336	0.034558466
TCONS_00162688	path:ko03460	0.021403902	0.040915995
TCONS_00054903	path:ko00563	0.021405192	0.049956277
TCONS_00175552	path:ko04550	0.0214442	0.039269201
TCONS_00059135	path:ko04152	0.021476252	0.042952503
TCONS_00111084	path:ko03013	0.02179419	0.037103305
TCONS_00111084	path:ko04213	0.021825474	0.037103305
TCONS_00180450	path:ko04114	0.021952311	0.044448631
TCONS_00040392	path:ko04064	0.021954987	0.031632002
TCONS_00008583	path:ko00564	0.02205463	0.039226943
TCONS_00001295	path:ko04111	0.02223476	0.04361725
TCONS_00066898	path:ko04962	0.022378274	0.035805238
TCONS_00019890	path:ko03015	0.022385542	0.046396193
TCONS_00028509	path:ko04360	0.022387235	0.039109544
TCONS_00168834	path:ko04810	0.022437382	0.031895438
TCONS_00175552	path:ko00563	0.022439544	0.039269201
TCONS_00089741	path:ko04726	0.022444939	0.039608715
TCONS_00089751	path:ko04350	0.022555764	0.029000268
TCONS_00162688	path:ko00563	0.022615191	0.040915995
TCONS_00008583	path:ko04660	0.022710336	0.039226943
TCONS_00028509	path:ko04510	0.022797055	0.039109544
TCONS_00028510	path:ko04919	0.022887551	0.0472033
TCONS_00162688	path:ko04071	0.023015247	0.040915995
TCONS_00040392	path:ko04922	0.023196802	0.031632002
TCONS_00028509	path:ko04725	0.023236079	0.039109544
TCONS_00089035	path:ko00270	0.023257521	0.045292376
TCONS_00111709	path:ko04370	0.02332178	0.035243248
TCONS_00152371	path:ko00020	0.023343668	0.03769667
TCONS_00028509	path:ko04011	0.023465726	0.039109544
TCONS_00028510	path:ko04360	0.023573359	0.0472033
TCONS_00066324	path:ko04120	0.023634515	0.044355285
TCONS_00180450	path:ko04152	0.023645917	0.044448631
TCONS_00053949	path:ko04921	0.023653346	0.037081412
TCONS_00180450	path:ko04720	0.02369265	0.044448631
TCONS_00168834	path:ko00260	0.023769997	0.031895438
TCONS_00066324	path:ko04130	0.023777174	0.044355285
TCONS_00175552	path:ko04726	0.023910904	0.039382665

TCONS_00180450	path:ko04024	0.023933878	0.044448631
TCONS_00152371	path:ko04626	0.024008244	0.03769667
TCONS_00181075	path:ko00010	0.024160774	0.03534546
TCONS_00001295	path:ko04722	0.024231806	0.04361725
TCONS_00108000	path:ko03050	0.024255822	0.036383733
TCONS_00111709	path:ko04730	0.024376188	0.035243248
TCONS_00000678	path:ko04139	0.024377377	0.03810127
TCONS_00089741	path:ko00532	0.024427773	0.040712954
TCONS_00053949	path:ko04060	0.024435592	0.037081412
TCONS_00168834	path:ko00670	0.024534952	0.031895438
TCONS_00028509	path:ko00230	0.024929612	0.039362546
TCONS_00008583	path:ko00562	0.02500144	0.039585613
TCONS_00054903	path:ko04310	0.02503272	0.049956277
TCONS_00152371	path:ko00010	0.025131114	0.03769667
TCONS_00111709	path:ko04611	0.025173749	0.035243248
TCONS_00000678	path:ko00051	0.025435699	0.03810127
TCONS_00019890	path:ko04916	0.025612623	0.046396193
TCONS_00089035	path:ko03015	0.025629777	0.045292376
TCONS_00181075	path:ko04745	0.025793517	0.03534546
TCONS_00019890	path:ko04728	0.025801646	0.046396193
TCONS_00162688	path:ko04391	0.025806271	0.041124077
TCONS_00107469	path:ko04210	0.02583605	0.042046445
TCONS_00059135	path:ko00900	0.025849656	0.045236898
TCONS_00054903	path:ko04211	0.025857226	0.049956277
TCONS_00053949	path:ko04920	0.026025898	0.037081412
TCONS_00157353	path:ko04550	0.026174689	0.045002043
TCONS_00033508	path:ko04514	0.026563106	0.045127218
TCONS_00175552	path:ko04611	0.0265963	0.041067843
TCONS_00111084	path:ko04212	0.026607586	0.04118166
TCONS_00135735	path:ko04070	0.026665744	0.046664796
TCONS_00000679	path:ko00510	0.026719105	0.045130645
TCONS_00028509	path:ko03018	0.02672492	0.039966246
TCONS_00111709	path:ko04966	0.026774455	0.035699273
TCONS_00181075	path:ko03022	0.026902515	0.03534546
TCONS_00111084	path:ko04966	0.026922041	0.04118166
TCONS_00107469	path:ko04011	0.027036377	0.042046445
TCONS_00059692	path:ko00620	0.027050319	0.036614976
TCONS_00059692	path:ko00750	0.027097875	0.036614976
TCONS_00019890	path:ko00523	0.027187748	0.046396193
TCONS_00019890	path:ko01055	0.027187748	0.046396193
TCONS_00135735	path:ko01200	0.027233208	0.046664796
TCONS_00099739	path:ko04966	0.027273304	0.037500794
TCONS_00053949	path:ko04071	0.027345758	0.037081412
TCONS_00059692	path:ko04391	0.027461232	0.036614976
TCONS_00000678	path:ko00533	0.027517584	0.03810127
TCONS_00181075	path:ko04915	0.027661664	0.03534546
TCONS_00000679	path:ko04145	0.027799997	0.045130645

TCONS_00053949	path:ko01210	0.027809764	0.037081412
TCONS_00111084	path:ko00603	0.027858182	0.04118166
TCONS_00157353	path:ko04670	0.027962394	0.045002043
TCONS_00083441	path:ko00604	0.027964652	0.042058834
TCONS_00028509	path:ko04711	0.027976372	0.039966246
TCONS_00089741	path:ko04713	0.027990864	0.042635735
TCONS_00053949	path:ko04810	0.028174389	0.037081412
TCONS_00053949	path:ko04611	0.028181873	0.037081412
TCONS_00152371	path:ko04141	0.028319205	0.038667735
TCONS_00033508	path:ko03018	0.028354913	0.045127218
TCONS_00107469	path:ko00604	0.028531516	0.042046445
TCONS_00152371	path:ko04214	0.028642767	0.038667735
TCONS_00040392	path:ko04630	0.028662947	0.035828684
TCONS_00175552	path:ko04912	0.028823991	0.041067843
TCONS_00083441	path:ko04920	0.028832916	0.042058834
TCONS_00157353	path:ko00350	0.029103563	0.045002043
TCONS_00083441	path:ko00190	0.029117655	0.042058834
TCONS_00175552	path:ko04962	0.029334174	0.041067843
TCONS_00162688	path:ko04962	0.02961573	0.041124077
TCONS_00135735	path:ko04725	0.029952617	0.046664796
TCONS_00028510	path:ko04912	0.029956559	0.0472033
TCONS_00066898	path:ko00630	0.030016706	0.043741601
TCONS_00066898	path:ko04214	0.030072351	0.043741601
TCONS_00059692	path:ko04210	0.03024018	0.038707431
TCONS_00053949	path:ko04520	0.030255555	0.037819444
TCONS_00089741	path:ko04727	0.030267498	0.042635735
TCONS_00028510	path:ko03040	0.030289954	0.0472033
TCONS_00152371	path:ko04210	0.030317072	0.038979092
TCONS_00000678	path:ko04145	0.030369567	0.038693808
TCONS_00000679	path:ko04142	0.030612992	0.045130645
TCONS_00099925	path:ko04622	0.030772891	0.049553222
TCONS_00162688	path:ko04630	0.030843058	0.041124077
TCONS_00008583	path:ko04011	0.030859733	0.045102687
TCONS_00107469	path:ko04915	0.030872688	0.043221764
TCONS_00180450	path:ko04080	0.03099729	0.048662283
TCONS_00168834	path:ko04145	0.031001376	0.035015922
TCONS_00099925	path:ko04710	0.031192478	0.049553222
TCONS_00135735	path:ko04745	0.031200058	0.046664796
TCONS_00028509	path:ko04921	0.031222352	0.042575934
TCONS_00000679	path:ko00720	0.03131669	0.045130645
TCONS_00066324	path:ko04662	0.03132526	0.044355285
TCONS_00111709	path:ko04211	0.03132607	0.037830436
TCONS_00059135	path:ko04020	0.031355546	0.048076372
TCONS_00089741	path:ko04370	0.031486341	0.042635735
TCONS_00116477	path:ko04130	0.031544853	0.042059804
TCONS_00135735	path:ko04150	0.031665397	0.046664796
TCONS_00108000	path:ko04966	0.03190667	0.044669339

TCONS_00028510	path:ko04664	0.031920108	0.0472033
TCONS_00066324	path:ko04925	0.031956128	0.044355285
TCONS_00111084	path:ko04660	0.032146811	0.044867426
TCONS_00066324	path:ko03410	0.032204372	0.044355285
TCONS_00000678	path:ko00720	0.03224484	0.038693808
TCONS_00089035	path:ko00523	0.032297025	0.045292376
TCONS_00089035	path:ko01055	0.032297025	0.045292376
TCONS_00168834	path:ko04510	0.032322389	0.035015922
TCONS_00111709	path:ko04916	0.032380027	0.037830436
TCONS_00089741	path:ko04910	0.032486869	0.042635735
TCONS_00111709	path:ko04068	0.032814022	0.037830436
TCONS_00066898	path:ko03013	0.032858774	0.044083683
TCONS_00180450	path:ko00521	0.032876349	0.048662283
TCONS_00111084	path:ko04013	0.032990755	0.044867426
TCONS_00175552	path:ko04924	0.033136052	0.044181403
TCONS_00152371	path:ko04012	0.033150145	0.040684268
TCONS_00028510	path:ko04713	0.033200739	0.0472033
TCONS_00001295	path:ko03018	0.033295681	0.047345479
TCONS_00099739	path:ko04144	0.033324788	0.040730296
TCONS_00001295	path:ko04670	0.033487592	0.047345479
TCONS_00089741	path:ko04921	0.033612016	0.042635735
TCONS_00019890	path:ko04113	0.033662651	0.046396193
TCONS_00111709	path:ko04728	0.033777175	0.037830436
TCONS_00089035	path:ko04350	0.033969282	0.045292376
TCONS_00089741	path:ko04915	0.034108588	0.042635735
TCONS_00028510	path:ko04145	0.034130351	0.0472033
TCONS_00059692	path:ko00630	0.034361859	0.041481103
TCONS_00066898	path:ko04662	0.034495663	0.044083683
TCONS_00028509	path:ko00750	0.034554814	0.0445192
TCONS_00059135	path:ko04080	0.034678223	0.048076372
TCONS_00157353	path:ko04145	0.034717448	0.045002043
TCONS_00040392	path:ko00260	0.034748625	0.038831867
